# Potato consumption and risk of cardio-metabolic diseases: evidence mapping of observational studies

**DOI:** 10.1186/s13643-020-01519-y

**Published:** 2020-12-01

**Authors:** Jisun So, Esther E. Avendano, Gowri Raman, Elizabeth J. Johnson

**Affiliations:** 1 Gerald J. and Dorothy R. Friedman School of Nutrition Science and Policy, Tufts University, Boston, MA 02111 USA; 2 Institute for Clinical Research and Health Policy Studies, Center for Clinical Evidence Synthesis, Tufts Medical Center, Boston, MA 02111 USA

**Keywords:** Potato, French fries, Cardiovascular disease, Diabetes, Hypertension, Stroke, Epidemiology, Dietary pattern

## Abstract

**Background:**

Recent systematic review of clinical trials concluded that there was no convincing evidence to suggest an association between potatoes and risk of cardio-metabolic diseases.

**Objective:**

Summarize observational study data related to potato intake and cardio-metabolic health outcomes in adults using evidence mapping to assess the need for a future systematic review.

**Methods:**

We searched MEDLINE®, Commonwealth Agricultural Bureau, and bibliographies for eligible observational studies published between 1946 and July 2020. Included studies evaluated potato intake in any form or as part of a dietary pattern with risk for cardio-metabolic diseases. Outcomes of interest included cardiovascular disease (CVD), cerebrovascular diseases, diabetes, hypertension, blood lipids, and body composition.

**Results:**

Of 121 eligible studies, 51 reported two different methods to quantify potato intake (30 studies quantified intake as either grams or serving; 20 studies reported times per week; one reported both methods) and 70 reported potato as part of a dietary pattern and compared higher vs. lower intake, linear change, or difference in potato intake among cases and controls. Studies that quantified potato intake as either grams or serving reported the following outcomes: diabetes (8 studies); cerebrovascular stroke (6 studies); five studies each for CVD, systolic and diastolic blood pressure, and hypertension; three studies each for body mass index, body weight, CVD mortality; two studies for myocardial infarction; and one study each for blood glucose, HOMA-IR, and blood lipids. Higher potato intake was associated with an increased risk for blood pressure and body weight, and the results of all other outcomes observed no association. Potato consumption as part of dietary pattern studies reported a negative association between fried form of potato and all or most cardio-metabolic risk factors and diseases.

**Conclusion:**

Evidence mapping found sufficient data on the association between potato intake and cardio-metabolic disease risk factors to warrant for a systematic review/meta-analysis of observational studies.

**Supplementary Information:**

The online version contains supplementary material available at 10.1186/s13643-020-01519-y.

## Background

Potatoes, a predominant food staple in the USA [1], contain a variety of nutrients and phytochemicals that include potassium, vitamin C, phosphorus, magnesium, B vitamins, dietary fiber, and polyphenols [2]. Potatoes contribute the third-highest total phenolic content to the diet among fruits and vegetables, after oranges and apples [2]. They are carbohydrate-rich providing little fat and many of the compounds found in potatoes have been shown to be beneficial to health through antioxidant, anti-inflammatory, and anti-hyperlipidemic actions [3]. In contrast, they are also considered to be a high-glycemic-index food [4] and consumption has been suggested to increase the cardio-metabolic risk of type 2 diabetes, obesity, and cardiovascular disease (CVD). However, a recent systematic review of clinical trials concluded that there was no convincing evidence to suggest an association between intake of potatoes and risk of these diseases [5]. Furthermore, results from single-meal test studies have found that intake of boiled potatoes increased satiety compared with intake of other iso-caloric preparations of rice, bread, and pasta [6].

The purpose of this evidence map is to summarize observational and epidemiologic studies examining potato intake and biomarkers of cardio-metabolic health with an objective to identify a comprehensive evidence base for conduct of further systematic review/meta-analysis.

## Methods

### Description of evidence maps

An evidence map is a systematic search of a broad field to organize, summarize, and synthesize current scientific evidence into a visual representation, often a tabular format or a searchable database [7]. Evidence mapping helps identify not only the areas rich in studies for the conduct of further systematic review or meta-analysis but also can identify gaps in knowledge for future research needs [8]. However, evidence mapping does not assess risk of bias or meta-analyze included studies. Our evidence map depicts a summary of literature on the relationship between potato intake and cardio-metabolic health outcomes and biomarkers of cardio-metabolic risk. Using the methodology outlined in the standard systematic review methods [9–11], we followed these steps: (1) identify the scope of search and the guiding question; (2) organize a team and assign each person’s roles and responsibilities; (3) develop a strategy for a systematic and comprehensive search; (4) define scientific criteria and approach to the selection of studies; (5) screen potentially eligible abstracts; (6) extract data during full-text screening; and (7) categorize the outcomes and summarize the characteristics of included studies. We constructed a study flow diagram to describe our flow of study screening and inclusion from the retrieved data. This is a review of published literature and therefore, it is not necessary to include a statement regarding adherence to the guidelines of the Declaration of Helsinki and Institutional Review Board approval.

### Identification of the scope

Our goal was to identify and summarize the extent and distribution of current evidence on the guiding key question that assessed whether higher potato intake, compared to lower intake, was associated with cardio-metabolic health outcomes and biomarkers of cardio-metabolic risk.

### Strategy of systematic search and study selection

We conducted an electronic search for studies that evaluated potato intake and cardio-metabolic health outcomes, published from 1946 to July 2020 in MEDLINE® and Commonwealth Agricultural Bureau (CAB) databases. We also searched the bibliography of prior systematic reviews and eligible studies for relevant studies. In electronic searches, we combined the National Library of Medicine’s Medical Subject Headings (MeSH) terms, keyword nomenclature, or text words for potatoes in combination with health-related terms (Additional Table S1). Searches were limited to observational and epidemiological studies conducted among adults (≥ 18 years of age). Additional eligibility criteria are detailed in Table 1. The titles and abstracts identified in the literature searches were screened independently by two team members and any conflicts were reviewed by all team members and resolved in weekly team meetings. Full-text publications for citations that met the inclusion criteria were retrieved and were also screened independently by two team members using the predefined eligibility criteria (Table 1). Any conflicts during full-text screening were also reviewed and resolved as a team.

**Table 1:** Inclusion and exclusion criteria

**Inclusion criteria**	
Study design	Cross-sectional Prospective cohort Retrospective cohort Nested case control
Study population	All adults (≥ 18 years of age), healthy or at risk of chronic disease
Exposure	Potatoes (regardless of preparation methods) French fries/potato chips Dietary patterns including any form of potato
Outcomes	Body weight or body mass index Waist circumference Hyperlipidemia/blood lipid profile Diabetes/blood glucose levels/insulin resistance/hemoglobin A1c Hypertension/blood pressure Inflammatory markers Oxidative stress Metabolic syndrome Stroke or cerebrovascular diseases Cardiovascular disease Cardiovascular mortality
**Exclusion criteria**	
Study design	Randomized controlled trials Case reports
Study population	Children Adolescents
Outcomes	All-cause mortality Cancers Diseases that are not related to cardio-metabolic health

### Data extraction and synthesis

We collected pertinent data from eligible studies into the Systemic Review Data Repository (SRDR^TM^), a publicly available web-based database application. The basic components of data extraction included (1) population; (2) potato source; (3) study design (observational studies, including prospective cohorts and case-control design); (4) outcome; (5) duration of follow-up; (6) number of participants; (7) number of studies per outcome and exposure; and (8) funding source. We extracted data when analyses stratified data by potato type and/or by sex. One team member extracted pertinent data from the studies that met the inclusion criteria and a second team member verified the data entries. Any conflicts during the extraction phase were discussed by the assigned extractor and reviewer, and updated by the extractor. Extracted data was analyzed using Microsoft Excel 2007©, and was summarized in narrative form, tables, and figures.

## Results

**Fig. 1:**
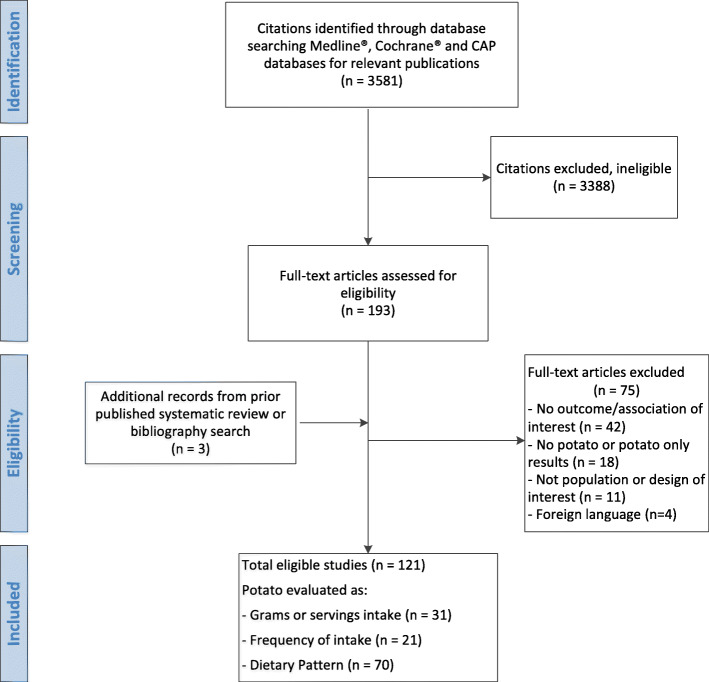
Study flow diagram. This figure shows the number of abstracts identified (*n* = 3581); abstracts not meeting criteria (*n* = 3388); full-text articles retrieved (*n* = 193); full-text articles excluded after screening (*n* = 75); full-text articles added from gray literature search (*n* = 3); full-text articles meeting study eligibility criteria (*n* = 121); eligible studies reporting results by grams or servings per week (*n* = 31), frequency of intake (*n* = 21), and dietary patterns or score (*n* = 70)

Our search identified 3581 abstracts. After the title and abstract screening in duplicate, 193 citations were identified for full-text retrieval and review against eligibility criteria. After full-text screening, a total of 121 articles were eligible for inclusion. The full list of included and excluded articles is listed in Additional Tables S2-S4, respectively. The study flow diagram is depicted in Fig. 1.

### Study design characteristics

We categorized eligible studies depending on the details of potato intake—25 unique studies (in 31 publications) quantified potato intake as either grams or servings per day; 21 studies reported frequency (e.g., times per week) of potato intake, but did not provide data on grams or servings per day, with the exception of one study that reported both intake as frequency and servings per day; and the remaining 69 unique studies (in 70 publications) included potato intake as part of a dietary pattern or score.

### Studies that quantified potato intake in grams or servings per day

Studies that quantified potato intake either as grams or servings per day included 15 cohort studies, 8 cross-sectional studies, and 3 case-control studies, while the Nurses’ Health Study [NHS] contributed to a longitudinal study and a case-control study (Additional Table S5). Seven of 8 cross-sectional studies were cross-sectional evaluation of potato intake from cohort studies.

### Study and population characteristics

Findings from the 25 observational studies included were reported between 1993 and 2019 (Table 2). The 15 cohort studies enrolled between 1981 and 410,701 participants (89,716 to 5,942,912 person-years). The 8 cross-sectional studies included between 110 and 41,391 subjects, and the 3 case-control studies enrolled between 390 and 2658 subjects. Funding sources included government only (14 studies, 56%), academia only (2 studies, 8%), and multiple funding sources (7 studies, 28%), and 2 studies (8%) did not report funding sources.

**Table 2:** Baseline characteristics of 25 included populations

	Total	Cohort	Cross sectional	Case control
***N*** **studies**	25^a^	15^b^	8	3
**Mean age**	36-73.7	36-67 (*n* = 11)	43.6-73.7	53.3-64.7
**Mean % male**	14.5-68.5	18.7-56.9	14.5-58.8	53.1-68.5
**Mean BMI**	23-30.8	23-30 (*n* = 12)	23.6-30.8 (*n* = 5)	26.9-27.2 (*n* = 2)
***N*** **analyzed**	110-410,701	1981-410,701	110-41,391	390-2658
**Follow-up duration**	0-24 years	2-24 years	NA	NA
**Baseline health**				
Healthy	5	4	1	
Overweight/obese	5	1	3	1
At risk or with CVD	10	7	2	2
Not reported	5	3	2	
**Study country**				
Asia Pacific	2		2	
Europe	14	8	5	1
Middle East	2	1		1
USA	7	6	1	1
**Intervention type**				
Potatoes	11	5	5	1
Boiled	1			1
Fried	3	2	1	
Mixed source^c^	10	8	2	1
**Funding type**				
Academia	2	1		1
Government	14	9	4	2
Mixed source	7	4	3	
Not reported	2	1	1	

*BMI* body mass index, *CVD* cardiovascular disease, *N* number; *NA* not applicable

^a^NHS is reported for both longitudinal and case-control results, so it has only been counted once in total

^b^15 cohorts are in 20 publications

^c^Reported combined results of various potato preparations

The average age of study populations at baseline ranged between 36 and 73.7 years (Table 2). Four studies included 100% female and 2 studies included 100% male participants.

### Potato intake assessment

The studies listed in Additional Table S5 examined potato intake using validated questionnaires at various average doses ranging from 5.3 g per day to greater than 286 g per day, and from less than 1 serving per month to 1 serving per day. Studies utilized different types of comparisons—13 studies (52%) compared the highest quantile to the lowest quantile of potato intake, 16 studies (64%) compared the outcomes based on a linear change of potato intake, and 3 studies (12%) compared potato intake between those who had a cardio-metabolic disease and control subjects. Of these, 2 studies reported results from all three types of comparisons and 3 studies reported results by both quantiles and linear change.

### Outcome descriptions

Outcomes reported in the evaluated studies are listed in Additional Table S5. All included studies adjusted models for potential confounders including, sex and age, diet, or other risk factors of cardio-metabolic disorders.

#### Cardiovascular disease

The CVD-related outcomes reported were incidence of overall CVD (4 studies, 16%), stroke (4 studies, 16%), myocardial infarction (2 studies, 8%), and CVD deaths (3 studies, 12%). Most studies observed no difference in the outcomes except for one study that reported decreased CVD mortality with higher intake of potatoes.

#### Type 2 diabetes and glucose homeostasis

The incidence of type 2 diabetes was reported by 8 studies (32%), and blood glucose concentrations and homeostatic model assessment—insulin resistance (HOMA-IR) were reported by 1 study (4%) each. Of 8 studies that assessed type 2 diabetes incidence, 4 (50%) studies reported increased risk associated with potato intake, whereas 2 (25%) reported no difference and 2 (25%) reported a decreased risk. Potato intake was found to be associated with a higher glucose concentration but not with HOMA-IR.

#### Hypertension and blood pressure measures

Five studies (20%) assessed hypertension as an outcome, reporting no difference in incidence in 3 studies and an increased incidence in 2 studies. Systolic blood pressure (SBP) was measured in 5 studies (20%); 3 reported an increased SBP, 1 reported no difference, and 1 found decreased SBP with potato intake. Diastolic blood pressure (DBP) was also assessed in 5 studies (20%); 3 reported no difference, 1 reported an increase, and 1 reported a decrease in DBP with higher intake of potatoes.

#### Blood lipids

One study (4%) each, measured total cholesterol (TC), high-density lipoprotein cholesterol (HDL-C), low-density lipoprotein cholesterol (LDL-C), and triglycerides (TG) and found no difference between higher and lower intake of potatoes.

#### Body mass index and body weight

Six studies (24%) assessed either BMI (3 studies) or body weight (3 studies); of these five studies reported, an increased BMI or body weight with potato intake and one study found no association between potato intake and BMI.

#### Other outcomes

**Fig. 2:**
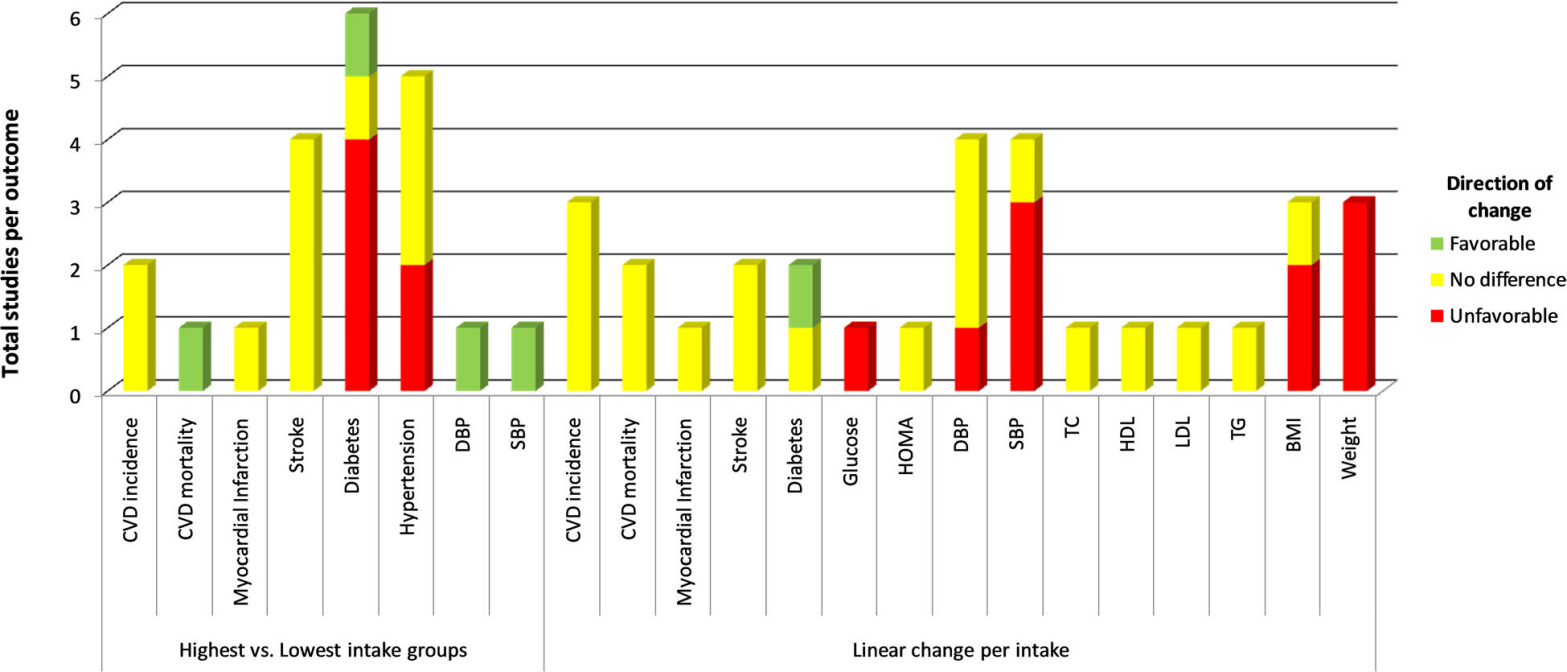
Association by type of analysis and per outcome in studies that quantified potato intake. This bar graph shows the number of studies reported for each outcome and the breakdown of statistical significance (green color indicates favorable association/decreased risk; yellow color indicates no difference; red color indicates unfavorable association/increased risk for cardio-metabolic outcomes). CVD, cardiovascular disease; DBP, diastolic blood pressure; SBP, systolic blood pressure; CHD, coronary heart disease; HOMA-IR, homeostatic model assessment—insulin resistance; TC, total cholesterol; HDL, high-density lipoprotein; LDL, low-density lipoprotein; TG, triglycerides; BMI, body mass index

One study (4%) measured the volume of visceral adipose tissue (VAT) and subcutaneous abdominal adipose tissue (SAAT) as outcomes, reporting no association with potato intake. One study (4%) assessed malondialdehyde (MDA) as a marker of oxidative stress and reported that higher intake of potatoes was associated with an increased level of MDA. One study (4%) reported change in hs-CRP, oxidized low-density lipoprotein, and Interleukin-6, and found that higher intake of deep-fried potatoes was associated with higher concentrations of hs-CRP, but not with oxidized low-density lipoprotein and IL-6 (Fig. 2).

### Studies with frequency information of potato intake

**Fig. 3:**
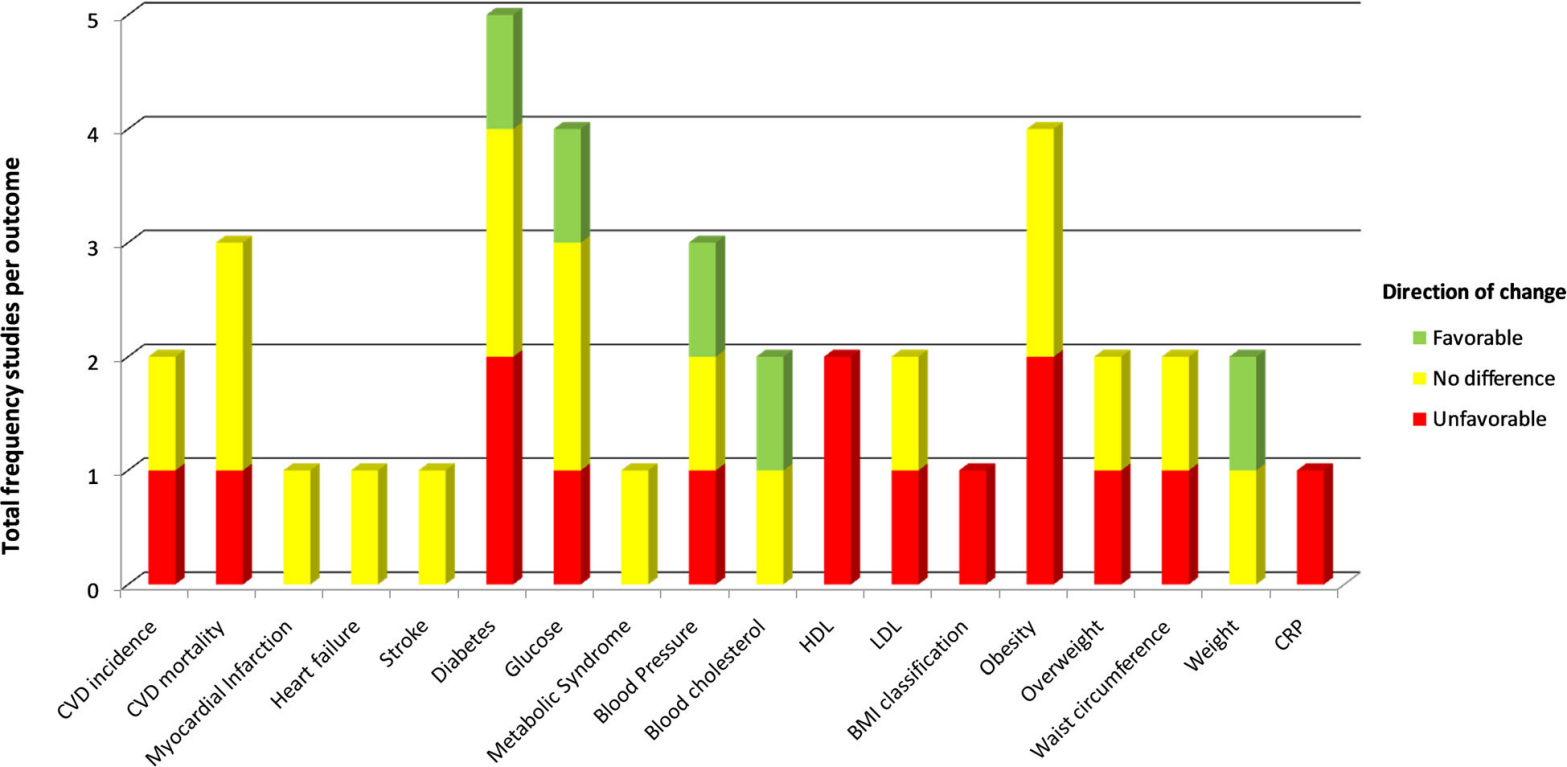
Association by outcome in studies that assessed potato intake as times per week. This bar graph shows the number of frequency studies reported for each outcome and the breakdown of statistical significance by color (green color indicates favorable association/decreased risk; yellow color indicates no difference; red color indicates unfavorable association/increased risk for cardio-metabolic outcomes). CVD, cardiovascular disease; HDL, high-density lipoprotein; LDL, low-density lipoprotein; BMI, body mass index; hs-CRP, high-sensitivity C-reactive protein

Among the 21 studies that reported potato intake by frequency of consumption (e.g., times per week), 10 were cohort studies with a follow-up duration ranged from 4 to 20 years and 11 were cross-sectional studies. The total number of participants enrolled was between 338 and 410,701 in the cohort studies and between 210 and 50,339 in the cross-sectional studies. Four studies (19%) included only male subjects while five studies (24%) enrolled only female subjects and the remaining studies included both males and females. The most frequently measured outcomes were the incidence of type 2 diabetes (5 studies, 24%), obesity (4 studies, 19%), and blood concentrations of glucose (3 studies, 14%) (Fig. 3).

### Outcomes in studies with potato intake frequency information

Potato intake increased the risk of the following outcomes: CVD (1 of 2 studies); CVD death (1 of 3 studies); incident diabetes (2 of 5 studies); impaired glucose tolerance (1 study); increased blood pressure among men (1 study); increased LDL-C levels (1 of 2 studies); obesity (2 of 4 studies); and increase in waist circumference among women (1 of 2 studies).

Favorable associations with potato intake were also found for the following outcomes: decreased risk of diabetes (1 study); a reduction in 2-h plasma glucose levels (1 study); and a decrease in body weight with boiled potatoes (1 study). All other remaining studies found no association between potato intake and outcomes.

### Studies reporting potato intake as dietary patterns

Of 69 studies in 70 publications that reported potato intake as a component of a dietary pattern or pre-defined dietary score, 57 studies reported on potatoes as part of a dietary pattern, 11 studies reported potatoes as part of a dietary score, and 1 study reported potato intake as both part of a dietary pattern and a dietary score. Forty-six studies assessed the overall intake of potato regardless of potato preparation type or failed to specify potato preparation type. Of these 46 studies that included potato in a dietary pattern, 26 studies reported a higher risk of cardio-metabolic disease, 11 studies reported a lower disease risk, 1 study that included potatoes in a prudent diet had lower risk, but higher risk in the western diet group along with French fries, and the remaining 8 studies reported no association. On the other hand, 26 remaining studies examined dietary patterns that specifically contained French fries or potato chips. In all but one study (with no association) were associated with incident and/or risk factors of cardio-metabolic disease. Of note, among all 69 studies, 3 studies reported results stratified by types of potato preparation, all 3 studies found an increase in cardio-metabolic risk factors (e.g., blood insulin, BMI) with dietary patterns that included French fries or potato chips, but not with the dietary pattern that included baked or boiled potatoes.

### Research-dense areas and evidence gaps

The most extensively evaluated outcomes were the incidence of CVD, type 2 diabetes, and obesity. However, we found less sufficient evidence for the associations between potato intake and biomarkers of cardio-metabolic risk (e.g., inflammation, oxidative stress, and blood lipids). For example, we have identified only two studies that reported the assessment of inflammation with C-reactive protein (CRP).

Regarding potato intake measured, we found a considerable heterogeneity in the way potato was prepared: boiled, fried, or both. However, the majority of the studies did not perform subgroup analyses, resulting in a lack of evidence assessing the influence of specific types of potato intake on cardio-metabolic risk.

## Discussions

This evidence map has identified a large body of epidemiological evidence that evaluated potato intake with cardio-metabolic disease outcomes and categorized outcome data according to the quantified methods of potato intake, as reported in individual studies. Among studies that quantified potato intake, there was an increased risk of type 2 diabetes, weight gain, and SBP, but not for clinical endpoints of CVD and CVD mortality. However, among studies that provided frequency information on potato intake, without specifying the quantity of potatoes consumed, the associations with overall CVD risk factors or outcomes were less conclusive. In studies of dietary pattern or score, dietary patterns that included French fries or potato chips were associated with an increased cardio-metabolic risk or CVD mortality. Notably, such associations were not observed for dietary patterns that contained non-fried types of potatoes (baked or boiled), which emphasizes that the fried types of potato preparations along with associated types of foods consumed with potatoes may increase the cardio-metabolic risk.

The most consistent results from studies that quantified potato intake found that an increased consumption of potatoes was generally related to an increased risk of weight gain and type 2 diabetes. For body weight, two cohorts reported by subgroups of potatoes (such as baked/boiled, fries, or chips) showed unfavorable outcomes, irrespective of the type of potato preparations, and one cohort that reported results for any type of potato also found an unfavorable outcome. This may be attributed to the high content of starches or refined carbohydrates in potatoes as it has been reported that the amount of starches or refined carbohydrates contained in foods was most associated with weight gain rather than other dietary metrics that are currently believed such as fat content or energy density [12]. Also, potatoes have glycemic index (GI) values in a relatively high range regardless of the cooking method [13]. For diabetes, most cohorts conducted in the USA reported unfavorable outcomes associated with total potato intake, whereas one study conducted in China and another conducted in Iran reported a favorable outcome for type 2 diabetes. It suggests that the dietary pattern may be of particular importance in evaluation of potato intake and health outcomes, considering that different types of potatoes are consumed in different cultures (e.g., mashed or fried potatoes in Western diet; boiled potatoes in Asian and Mediterranean diets) [14]. Although potatoes have been condemned as unhealthy due to high carbohydrate content and GI, they are rich in potassium, magnesium, vitamin C, B vitamins, fiber, and polyphenols [13–15], each of which is associated with a decreased risk of chronic disease. However, most often potatoes are not consumed alone [4] and therefore, it may be more important to assess the effects of potatoes along with the type of diet or dietary patterns rather than assessing the effects of potatoes on outcomes of interest. The different types of potato preparations are often co-consumed with different types of diet [16, 17]. For example, French fries are commonly served with fast foods (such as burgers and sodas), potato chips as a snack, and baked/boiled potatoes are part of a meal. The differences in co-consumed diets may explain the difference in our evidence mapping results. Further careful evaluations of dietary patterns may help understand the inconsistencies in the results across studies quantified potato intake.

### Description of existing literature

Two meta-analyses have been published on the association of potato intake with risk of chronic disease and mortality [14, 15]. A previous systematic review of 20 prospective cohort studies, which focused on various types of mortality as outcomes, reported no association between total potato intake and risk of all-cause and cancer mortality as well as insufficient evidence accumulated for CVD mortality [18]. However, another meta-analysis of 28 reports from prospective studies showed that a one daily serving (150 g/d) increase in total potato intake was associated with an 18% (95% CI, 10-27%) increase in risk for type 2 diabetes and 12% (95% CI, 1-23%) increase in risk for hypertension while reporting no association with risk of all-cause mortality, CHD, stroke, and colorectal cancer [19]. French fries consumption that was specified only in a smaller subset of the included studies showed a stronger positive association with type 2 diabetes (RR, 1.66; 95% CI, 1.43-1.94) and hypertension (RR, 1.37; 95% CI, 1.15-1.63) risks. A meta-analysis of six cohort studies showed that an increase of one daily serving of total potato intake was associated with a 20% (95% CI, 13-27%) increase in risk of type 2 diabetes [20]. More recently, one large-scale meta-analysis of 185 prospective studies and 58 RCTs was published on the relationship between carbohydrate quality (i.e., dietary fiber, glycemic index/load, and whole-grain intake) and chronic disease outcomes; however, no specific data was available on potato intake [21].

### Strengths and limitations

Increasingly, evidence mapping methods are currently used to identify gaps and topics for future systematic reviews. Our review identified a variety of epidemiological methods used in evaluation of potato intake and chronic disease outcomes. The strengths of our approach include evaluation of different types of observational designs as well as the different types of evaluations (quantity of potato intake versus dietary pattern studies). Our review identified that there are sufficient number of studies available for conducting a future systematic review.

The limitation of evidence mapping includes lack of critical appraisal of individual study quality. Observational studies using food frequency questionnaires are often limited by the participants recall bias. Nonetheless, among eligible studies, there was considerable heterogeneity regarding potato intake; they were consumed as boiled, fried, or both and studies often failed to report subgroup analyses by types of potato preparation. Among studies that quantified potato, only a few cohorts contributed to the majority of the data. This may impact the generalizability to larger populations. Studies that provided intake data in terms of frequency per week intake of potato had incomplete information on the total intake per week or per day, precluding their utilization in future meta-analysis to assess a dose-response relationship. Although we included only observational studies, there was heterogeneity in the way outcome data was reported with some studies reporting results for longitudinal data, while others reported results for cross-sectional or case controls.

## Conclusion

Our qualitative gap analysis using evidence mapping has identified 121 different observational studies including prospective cohort, case-control, and cross-sectional studies to examine if higher potato intake is associated with an increased risk of developing cardio-metabolic disease as well as higher CVD risk factors. Our findings demonstrate sufficient evidence on the relationship between potato intake and risk factors associated with CVD such as type 2 diabetes, weight gain, and high blood pressure in particular. This evidence map also identifies ample evidence available for a future systematic review and meta-analysis for these outcomes, addressing the need for a thorough evaluation of different types of potato preparations as well as the accompanied diet.

## Supplementary Information

**Additional file 1: Table S1.** Search strategy. Table listing the terms used to search MEDLINE® and Commonwealth Agricultural Bureau databases. **Table S2.** List of 51 articles included in Evidence Map. List of studies articles included in the evidence map. **Table S3.** List of 69 Unique Dietary Pattern or Dietary Score studies included in Evidence Map. List of dietary pattern or dietary score articles included in the evidence map. **Table S4.** List of 75 studies identified from abstract screening but excluded after full-text screening. List of studies excluded during full-text screening. **Table S5.** Baseline characteristics of included studies. Table listing the cohort names, study design, enrollment years, country, funding, N analyzed, reported age of participants, percent of male participants, baseline health status of participants, type of potato consumed, type of analysis, potato intake amounts, and list of study outcomes reported for each study.

## Data Availability

The dataset supporting the conclusions of this article is included within the article and its additional tables and figures.

## Abbreviations

BMI: Body mass index
CRP: C-reactive protein
CVD: Cardiovascular disease
DBP: Diastolic blood pressure
HDL: High-density lipoprotein
HOMA-IR: Homeostatic model assessment—insulin resistance
LDL: Low-density lipoprotein
MDA: Malondialdehyde
MeSH: Medical Subject Headings
NHS: Nurses’ Health Study
SAAT: Subcutaneous abdominal adipose tissue
SBP: Systolic blood pressure
SRDR: Systematic Review Data Repository
TC: Total cholesterol
TG: Triglyceride
USA: United States of America
USDA: United States Department of Agriculture
VAT: Visceral adipose tissue
