# Supplementary material for: Potato consumption and risk of cardio-metabolic diseases: evidence mapping of observational studies
Source: Syst Rev. 2020 Dec 1;9:274. doi: 10.1186/s13643-020-01519-y (PMC7706195; doi:10.1186/s13643-020-01519-y)
Supplement: Supplementary file 1 — Additional file 1: Table S1. Search strategy. Table listing the terms used to search MEDLINE® and Commonwealth Agricultural Bureau databases. Table S2. List of 51 articles included in Evidence Map. List of studies articles included in the evidence map. Table S3. List of 69 Unique Dietary Pattern or Dietary Score studies included in Evidence Map. List of dietary pattern or dietary score articles included in the evidence map. Table S4. List of 75 studies identified from abstract screening but excluded after full-text screening. List of studies excluded during full-text screening. Table S5. Baseline characteristics of included studies. Table listing the cohort names, study design, enrollment years, country, funding, N analyzed, reported age of participants, percent of male participants, baseline health status of participants, type of potato consumed, type of analysis, potato intake amounts, and list of study outcomes reported for each study. [file 13643_2020_1519_MOESM1_ESM.docx]

**Additional Table S1** Search strategy

| 1 | potato$.af. or exp Solanumtuberosum/ |
| --- | --- |
| 2 | (french adj1 fries).af. |
| 3 | exp cohort studies/ or exp prospective studies/ or exp retrospective studies/ or exp epidemiologic studies/ or exp case-control studies/ |
| 4 | (cohort or retrospective or prospective or longitudinal or observational or follow-up or followup or registry).af. |
| 5 | case-control.af. or (case adj10 control).tw. |
| 6 | ep.fs. |
| 7 | (cross-sectional or prevalence).tw. |
| 8 | (cross adj1 sectional).tw. |
| 9 | 3 or 4 or 5 or 6 or 7 or 8 |
| 10 | 1 or 2 |
| 11 | 9 and 10 |
| 12 | (Animal not human).mp. [mp=title, abstract, original title, name of substance word, subject heading word, floating sub-heading word, keyword heading word, protocol supplementary concept word, rare disease supplementary concept word, unique identifier, synonyms] |
| 13 | 11 not 12 |
| 14 | limit 13 to "all child (0 to 18 years)" |
| 15 | limit 13 to "all adult (19 plus years)" |
| 16 | 14 and 15 |
| 17 | 13 not 14 |
| 18 | 16 or 17 |

**Additional Table S2** List of 51 articles included in Evidence Map

| **PMID** | **Title** | **Author** | **Journal** | **Year** |
| --- | --- | --- | --- | --- |
| None | Prevalence of obesity among Saudi Board residents in Aseer Region, Saudi Arabia | Alzahrani AA, Al-Khaldi YM, lsamghan AS | Saudi Journal of Obesity | 2016 |
| 8621198 | Prospective study of nutritional factors, blood pressure, and hypertension among US women. | Ascherio A, Hennekens C, Willett WC, Sacks F, Rosner B, Manson J, Witteman J, Stampfer MJ. | Hypertension | 1996 |
| 27189229 | Potato intake and incidence of hypertension: results from three prospective US cohort studies. | Borgi L, Rimm EB, Willett WC, Forman JP. | BMJ | 2016 |
| None | Lifestyle correlates of overweight and obesity among the population of Ukraine | Chagarna NS, Andreeva TI | Tobacco Control and Public Health in Eastern Europe | 2014 |
| 29695838 | Leukocyte telomere length and diet in the apparently healthy, middle-aged Asklepios population | De Meyer T, Bekaert S, De Buyzere ML, De Bacquer DD, Langlois MR, Shivappa N, Hébert JR, Gillebert TC, Rietzschel ER, Huybrechts I | Sci Rep. | 2018 |
| 22894912 | Mediterranean diet and CHD: the Greek European Prospective Investigation into Cancer and Nutrition cohort. | Dilis V, Katsoulis M, Lagiou P, Trichopoulos D, Naska A, Trichopoulou A. | Br J Nutr | 2012 |
| 29909965 | The Association of Potato Intake With Risk for Incident Type 2 Diabetes in Adults | Farhadnejad H, Teymoori F, Asghari G, Mirmiran P, Azizi F | Can J Diabetes | 2018 |
| 7587845 | Dietary factors determining diabetes and impaired glucose tolerance. A 20-year follow-up of the Finnish and Dutch cohorts of the Seven Countries Study. | Feskens EJ, Virtanen SM, Rasanen L, Tuomilehto J, Stengard J, Pekkanen J, Nissinen A, Kromhout D. | Diabetes Care | 1995 |
| 8186811 | Predictors of weight change over two years among a population of working adults: the Healthy Worker Project. | French SA, Jeffery RW, Forster JL, McGovern PG, Kelder SH, Baxter JE. | Int J ObesRelatMetabDisord | 1994 |
| 27680091 | Food consumption and the actual statistics of cardiovascular diseases: an epidemiological comparison of 42 European countries | Grasgruber P, Sebera M, Hrazdira E, Hrebickova S, Cacek J | Food & Nutrition Research | 2016 |
| 15522143 | Food and drinking patterns as predictors of 6-year BMI-adjusted changes in waist circumference | HalkjaerJ, SorensenTI,Tjonneland A, Togo P, Holst C, Heitmann BL | British Journal of Nutrition | 2004 |
| 19631041 | Dietary predictors of 5-year changes in waist circumference. | Halkjaer J, Tjonneland A, Overvad K, Sorensen TI. | J Am Diet Assoc | 2009 |
| 16469985 | Potato and french fry consumption and risk of type 2 diabetes in women. | Halton TL, Willett WC, Liu S, Manson JE, Stampfer MJ, Hu FB. | Am J ClinNutr | 2006 |
| 31063480 | Potato consumption and the risk of overall and cause specific mortality in the NIH-AARP study | Hashemian M, Murphy G, Etemadi A, Liao L, Dawsey S, Malekzadeh R, Abnet C | PLoS One | 2019 |
| 27851788 | Dietary Information Improves Model Performance and Predictive Ability of a Noninvasive Type 2 Diabetes Risk Model. | Han T, Tian S, Wang L, Liang X, Cui H, Du S, Na G, Na L, Sun C | PLoS One | 2016 |
| 18329089 | Are the associations between diet and C-reactive protein independent of obesity? | Hickling S, Hung J,Knuiman M, Divitini M, BeilbyJ | Preventive Medicine | 2008 |
| 15505008 | Glycemic index and dietary fiber and the risk of type 2 diabetes. | Hodge AM, English DR, O'Dea K, Giles GG. | Diabetes Care | 2004 |
| 29046405 | Potato Consumption Does Not Increase Blood Pressure or Incident Hypertension in 2 Cohorts of Spanish Adults. | Hu EA, Martinez-Gonzalez MA, Salas-Salvado J, Corella D, Ros E, Fito M, Garcia-Rodriguez A, Estruch R, Aros F, Fiol M, Lapetra J, Serra-Majem L, Pinto X, Ruiz-Canela M, Razquin C, Bullo M, Sorli­ JV, Schrader H, Rebholz CM, Toledo E; PREDIMED Study and SUN Project Investigators. | J Nutr | 2017 |
| 11412050 | The effect of fruit and vegetable intake on risk for coronary heart disease. | Joshipura KJ, Hu FB, Manson JE, Stampfer MJ, Rimm EB, Speizer FE, Colditz G, Ascherio A, Rosner B, Spiegelman D, Willett WC. | Ann Intern Med | 2001 |
| 10517425 | Fruit and vegetable intake in relation to risk of ischemic stroke. | Joshipura KJ, Ascherio A, Manson JE, Stampfer MJ, Rimm EB, Speizer FE, Hennekens CH, Spiegelman D, Willett WC. | JAMA | 1999 |
| 25600675 | [Factors associated with six-year weight change in young and middle-aged adults in the Young Finns Study.](https://www.ncbi.nlm.nih.gov/pubmed/25600675) | Kaikkonen JE, Mikkilä V, Juonala M, Keltikangas-Järvinen L, Hintsanen M, Pulkki-Råback L, Viikari JS, Kähönen M, Lehtimäki T, Telama R, Raitakari OT. | Scand J Clin Lab Invest. | 2015 |
| 25188828 | [Prevalence and association of female weight status and dietary habits with sociodemographic factors: a cross-sectional study in Saudi Arabia.](https://www.ncbi.nlm.nih.gov/pubmed/25188828) | Khalaf A, Westergren A, Berggren V, Ekblom Ö, Al-Hazzaa HM. | Public Health Nutr. | 2015 |
| 22639829 | [Potato consumption and cardiovascular disease risk factors among Iranian population.](https://www.ncbi.nlm.nih.gov/pubmed/22639829) | Khosravi-Boroujeni H, Mohammadifard N, Sarrafzadegan N, Sajjadi F, Maghroun M, Khosravi A, Alikhasi H, Rafieian M, Azadbakht L. | Int J Food SciNutr. | 2012 |
| 23432170 | A case-control study on potato consumption and risk of stroke in central Iran. | Khosravi-Boroujeni H, Saadatnia M, Shakeri F, Keshteli AH, Esmaillzadeh A. | Arch Iran Med | 2013 |
| No PMID | A long term observation of total cholesterol, blood pressure, BMI and blood glucose concerned with dietary intake. | Kim IS and Seo EA | Korean J Community Nutrition | 2000 |
| 31882021 | Longitudinal association of changes in diet with changes in body weight and waist circumference in subjects at high cardiovascular risk: the PREDIMED trial | [Konieczna J](https://www.ncbi.nlm.nih.gov/pubmed/?term=Konieczna%20J%5BAuthor%5D&cauthor=true&cauthor_uid=31882021), [Romaguera D](https://www.ncbi.nlm.nih.gov/pubmed/?term=Romaguera%20D%5BAuthor%5D&cauthor=true&cauthor_uid=31882021), [Pereira V](https://www.ncbi.nlm.nih.gov/pubmed/?term=Pereira%20V%5BAuthor%5D&cauthor=true&cauthor_uid=31882021), [Fiol M](https://www.ncbi.nlm.nih.gov/pubmed/?term=Fiol%20M%5BAuthor%5D&cauthor=true&cauthor_uid=31882021), [Razquin C](https://www.ncbi.nlm.nih.gov/pubmed/?term=Razquin%20C%5BAuthor%5D&cauthor=true&cauthor_uid=31882021), [Estruch R](https://www.ncbi.nlm.nih.gov/pubmed/?term=Estruch%20R%5BAuthor%5D&cauthor=true&cauthor_uid=31882021), [Asensio EM](https://www.ncbi.nlm.nih.gov/pubmed/?term=Asensio%20EM%5BAuthor%5D&cauthor=true&cauthor_uid=31882021), [Babio N](https://www.ncbi.nlm.nih.gov/pubmed/?term=Babio%20N%5BAuthor%5D&cauthor=true&cauthor_uid=31882021), [Fitó M](https://www.ncbi.nlm.nih.gov/pubmed/?term=Fit%C3%B3%20M%5BAuthor%5D&cauthor=true&cauthor_uid=31882021)^2,9^, [Gómez-Gracia E](https://www.ncbi.nlm.nih.gov/pubmed/?term=G%C3%B3mez-Gracia%20E%5BAuthor%5D&cauthor=true&cauthor_uid=31882021), [Ros E](https://www.ncbi.nlm.nih.gov/pubmed/?term=Ros%20E%5BAuthor%5D&cauthor=true&cauthor_uid=31882021), [Lapetra J](https://www.ncbi.nlm.nih.gov/pubmed/?term=Lapetra%20J%5BAuthor%5D&cauthor=true&cauthor_uid=31882021), [Arós F](https://www.ncbi.nlm.nih.gov/pubmed/?term=Ar%C3%B3s%20F%5BAuthor%5D&cauthor=true&cauthor_uid=31882021), [Serra-Majem L](https://www.ncbi.nlm.nih.gov/pubmed/?term=Serra-Majem%20L%5BAuthor%5D&cauthor=true&cauthor_uid=31882021), [Pintó X](https://www.ncbi.nlm.nih.gov/pubmed/?term=Pint%C3%B3%20X%5BAuthor%5D&cauthor=true&cauthor_uid=31882021), [Toledo E](https://www.ncbi.nlm.nih.gov/pubmed/?term=Toledo%20E%5BAuthor%5D&cauthor=true&cauthor_uid=31882021), [Sorlí JV](https://www.ncbi.nlm.nih.gov/pubmed/?term=Sorl%C3%AD%20JV%5BAuthor%5D&cauthor=true&cauthor_uid=31882021), [Bulló M](https://www.ncbi.nlm.nih.gov/pubmed/?term=Bull%C3%B3%20M%5BAuthor%5D&cauthor=true&cauthor_uid=31882021), [Schröder H](https://www.ncbi.nlm.nih.gov/pubmed/?term=Schr%C3%B6der%20H%5BAuthor%5D&cauthor=true&cauthor_uid=31882021), [Martínez-González MA](https://www.ncbi.nlm.nih.gov/pubmed/?term=Mart%C3%ADnez-Gonz%C3%A1lez%20MA%5BAuthor%5D&cauthor=true&cauthor_uid=31882021) | [Int J Behav Nutr Phys Act.](https://www.ncbi.nlm.nih.gov/pubmed/31882021) | 2019 |
| 27680993 | [Potato consumption and risk of cardiovascular disease: 2 prospective cohort studies.](https://www.ncbi.nlm.nih.gov/pubmed/27680993) | Larsson SC, Wolk A | Am J ClinNutr. | 2016 |
| 14576712 | Food habits are associated with lipid peroxidation in an elderly population. | Lasheras C, Gonzalez S, Huerta JM, Lombardia C, Ibanez R, Patterson AM, Fernandez S. | J Am Diet Assoc | 2003 |
| 15562224 | A prospective study of fruit and vegetable intake and the risk of type 2 diabetes in women. | Liu S, Serdula M, Janket SJ, Cook NR, Sesso HD, Willett WC, Manson JE, Buring JE. | Diabetes Care | 2004 |
| 19379545 | [Food intake and prevalence of obesity in Brazil: an ecological analysis.](https://www.ncbi.nlm.nih.gov/pubmed/19379545) | Lobato JC, Costa AJ, Sichieri R. | Public Health Nutr. | 2009 |
| 18854749 | Anthropometric and dietary determinants of blood pressure in over 7000 Mediterranean women: the European Prospective Investigation into Cancer and Nutrition-Florence cohort. | Masala G, Bendinelli B, Versari D, Saieva C, Ceroti M, Santagiuliana F, Caini S, Salvini S, Sera F, Taddei S, Ghiadoni L, Palli D. | J Hypertens | 2008 |
| 19646291 | Plant foods and the risk of cerebrovascular diseases: a potential protection of fruit consumption. | Mizrahi A, Knekt P, Montonen J, Laaksonen MA, Heliovaara M, JÃ¤rvinen R. | Br J Nutr | 2009 |
| 15674312 | Food consumption and the incidence of type II diabetes mellitus. | Montonen J, Jarvinen R, Heliovaara M, Reunanen A, Aromaa A, Knekt P. | Eur J ClinNutr | 2005 |
| 24833275 | [Central obesity is associated with lower intake of whole-grain bread and less frequent breakfast and lunch: results from the HUNT study, an adult all-population survey.](https://www.ncbi.nlm.nih.gov/pubmed/24833275) | Mostad IL, Langaas M, Grill V. | ApplPhysiolNutrMetab. | 2014 |
| 21696306 | Changes in diet and lifestyle and long-term weight gain in women and men. | Mozaffarian D, Hao T, Rimm EB, Willett WC, Hu FB. | N Engl J Med | 2011 |
| 26681722 | Potato Consumption and Risk of Type 2 Diabetes: Results From Three Prospective Cohort Studies. | Muraki I, Rimm EB, Willett WC, Manson JE, Hu FB, Sun Q. | Diabetes Care | 2016 |
| 25415630 | [Socio-demographic and dietary factors associated with obesity among female university students in Jordan.](https://www.ncbi.nlm.nih.gov/pubmed/25415630) | Musaiger AO, Hammad SS, Tayyem RF, Qatatsheh AA. | Int J Adolesc Med Health. | 2015 |
| 8941095 | Intake of dietary fiber and risk of coronary heart disease in a cohort of Finnish men. The Alpha-Tocopherol, Beta-Carotene Cancer Prevention Study. | Pietinen P, Rimm EB, Korhonen P, Hartman AM, Willett WC, Albanes D, Virtamo J. | Circulation | 1996 |
| 16909460 | [Body mass index and nutrition as determinants of health and disease in population of Croatian Adriatic islands.](https://www.ncbi.nlm.nih.gov/pubmed/16909460) | Pucarin-Cvetković J, Mustajbegović J, DokoJelinić J, Senta A, Nola IA, Ivanković D, Kaić-Rak A, Milosević M. | Croat Med J. | 2006 |
| 26439793 | Association of food consumption with total volumes of visceral and subcutaneous abdominal adipose tissue in a Northern German population. | Ruttgers D, Fischer K, Koch M, Lieb W, Muller HP, Jacobs G, Kassubek J, Nothlings U. | Br J Nutr | 2015 |
| 9020271 | [Dietary fiber, glycemic load, and risk of non-insulin-dependent diabetes mellitus in women.](https://www.ncbi.nlm.nih.gov/pubmed/9020271) | Salmerón J, Manson JE, Stampfer MJ, Colditz GA, Wing AL, Willett WC. | JAMA. | 1997 |
| 26417841 | [Predicted 10-year risk of cardiovascular disease among Canadian adults using modified Framingham Risk Score in association with dietary intake.](https://www.ncbi.nlm.nih.gov/pubmed/26417841) | Setayeshgar S, Whiting SJ, Pahwa P, Vatanparast H. | ApplPhysiolNutrMetab. | 2015 |
| 27930484 | Are Hospital Workers Healthy?: A Study of Cardiometabolic, Behavioral, and Psychosocial Factors Associated With Obesity Among Hospital Workers. | Sharma SV, Upadhyaya M, Karhade M, Baun WB, Perkison WB, Pompeii LA, Brown HS, Hoelscher DM. | J Occup Environ Med | 2016 |
| 8269793 | Comparison of diets of diabetic and nondiabetic women. | Shimakawa T, Herrera-Acena MG, Colditz GA, Manson JE, Stampfer MJ, Willett WC, Stamper MJ. | Diabetes Care | 1993 |
| 25898210 | The association between carbohydrate-rich foods and risk of cardiovascular disease is not modified by genetic susceptibility to dyslipidemia as determined by 80 validated variants. | Sonestedt E, Hellstrand S, Schulz CA, Wallstrom P, Drake I, Ericson U, Gullberg B, Hedblad B, Orho-Melander M. | PLoS One | 2015 |
| 12807839 | Carbohydrates, dietary glycaemic load and glycaemic index, and risk of acute myocardial infarction. | Tavani A, Bosetti C, Negri E, Augustin LS, Jenkins DJ, La Vecchia C. | Heart | 2003 |
| 11338129 | Is there a different dietetic pattern depending on self-knowledge of high blood pressure? | Tormo MJ, Navarro C, Chirlaque MD, Barber X; EPIC Group of Spain. European Prospective Investigation on Cancer. | Eur J Epidemiol | 2000 |
| 18039989 | Prospective study of dietary carbohydrates, glycemic index, glycemic load, and incidence of type 2 diabetes mellitus in middle-aged Chinese women | Villegas R, Liu S, Gao Y, Yang G, Li H, Zheng W, Ou Shu X. | Arch Intern Med | 2007 |
| 23388667 | Diet and risk of chronic diseases: results from the first 8 years of follow-up in the EPIC-Potsdam study. | von Ruesten A, Feller S, Bergmann MM, Boeing H. | Eur J ClinNutr | 2013 |
| 10235173 | [Frequent salad vegetable consumption is associated with a reduction in the risk of diabetes mellitus.](https://www.ncbi.nlm.nih.gov/pubmed/10235173) | Williams DE, Wareham NJ, Cox BD, Byrne CD, Hales CN, Day NE. | J ClinEpidemiol. | 1999 |
| 27774916 | Substitution of meat and fish with vegetables or potatoes and risk of myocardial infarction. | Wurtz AM, Hansen MD, TjÃ¸nneland A, Rimm EB, Schmidt EB, Overvad K, Jakobsen MU. | Br J Nutr | 2016 |

**Additional Table S3** List of 69 Unique Dietary Pattern or Dietary Score studies included in Evidence Map

| **PMID** | **Title** | **Author** | **Journal** | **Year** |
| --- | --- | --- | --- | --- |
| 20624672 | Relationship between major dietary patterns and metabolic syndrome among individuals with impaired glucose tolerance. | Amini M, Esmaillzadeh A, Shafaeizadeh S, Behrooz J, Zare M. | Nutrition | 2010 |
| 27620002 | Dietary patterns and the risk of CVD and all-cause mortality in older British men. | Atkins JL, Whincup PH, Morris RW, Lennon LT, Papacosta O, Wannamethee SG. | Br J Nutr | 2016 |
| 16115339 | Dietary patterns among older Europeans: the EPIC-Elderly study. | Bamia C, Orfanos P, Ferrari P, Overvad K, Hundborg HH, Tjonneland A, Olsen A, Kesse E, Boutron-Ruault MC, Clavel-Chapelon F, Nagel G, Boffetta P, Boeing H, Hoffmann K, Trichopoulos D, Baibas N, Psaltopoulou T, Norat T, Slimani N, Palli D, Krogh V, Panico S, et al. | Br J Nutr | 2005 |
| 28378296 | Associations between dietary patterns, socio-demographic factors and anthropometric measurements in adult New Zealanders: an analysis of data from the 2008/09 New Zealand Adult Nutrition Survey. | Beck KL, Jones B, Ullah I, McNaughton SA, Haslett SJ, Stonehouse W. | Eur J Nutr. | 2018 |
| 21677127 | Associations between dietary patterns and LDL peak particle diameter: a cross-sectional study. | Bouchard-Mercier A, Paradis AM, Godin G, Lamarche B, Perusse L, Vohl MC. | J Am CollNutr | 2010 |
| 19744354 | Dietary patterns associated with metabolic syndrome, sociodemographic and lifestyle factors in young adults: the Bogalusa Heart Study. | Deshmukh-Taskar PR, O'Neil CE, Nicklas TA, Yang SJ, Liu Y, Gustat J, Berenson GS. | Public Health Nutr | 2009 |
| 19710163 | Dietary patterns are associated with metabolic syndrome in adult Samoans. | DiBello JR, McGarvey ST, Kraft P, Goldberg R, Campos H, Quested C, Laumoli TS, Baylin A. | J Nutr | 2009 |
| 27557817 | Dietary patterns in an elderly population and their relation with bone mineral density: the Rotterdam Study. | de Jonge EAL, Rivadeneira F, Erler NS, Hofman A, Uitterlinden AG, Franco OH, Kiefte-de Jong JC. | Eur J Nutr. | 2018 |
| 29141668, 29217921 | Association of dietary patterns with diabetes complications among type 2 diabetes patients in Gaza Strip, Palestine: a cross sectional study. | El Bilbeisi AH, Hosseini S, Djafarian K. | J Health PopulNutr | 2017 |
| 17374666 | Dietary patterns and markers of systemic inflammation among Iranian women. | Esmaillzadeh A, Kimiagar M, Mehrabi Y, Azadbakht L, Hu FB, Willett WC. | J Nutr | 2007 |
| 26070830 | Association between dietary patterns and metabolic syndrome in a sample of Tehranian adults. | Farhangi MA, Jahangiry L, Asghari-Jafarabadi M, Najafi M. | Obes Res ClinPract | 2016 |
| 16002815 | Diet-quality scores and plasma concentrations of markers of inflammation and endothelial dysfunction. | Fung TT, McCullough ML, Newby PK, Manson JE, Meigs JB, Rifai N, Willett WC, Hu FB. | Am J ClinNutr | 2005 |
| 15534160 | Dietary patterns, meat intake, and the risk of type 2 diabetes in women. | Fung TT, Schulze M, Manson JE, Willett WC, Hu FB. | Arch Intern Med | 2004 |
| 11124751 | Association between dietary patterns and plasma biomarkers of obesity and cardiovascular disease risk. | Fung TT, Rimm EB, Spiegelman D, Rifai N, Tofler GH, Willett WC, Hu FB. | Am J ClinNutr | 2001 |
| 11493127 | Dietary patterns and the risk of coronary heart disease in women. | Fung TT, Willett WC, Stampfer MJ, Manson JE, Hu FB. | Arch Intern Med | 2001 |
| 29695705 | Dietary patterns and type 2 diabetes among Ghanaian migrants in Europe and their compatriots in Ghana: the RODAM study. | Galbete C, Nicolaou M, Meeks K, Klipstein-Grobusch K, de-Graft Aikins A, Addo J, Amoah SK, Smeeth L, Owusu-Dabo E, Spranger J, Agyemang C, Mockenhaupt FP, Beune E, Stronks K, Schulze MB, Danquah I. | Nutr Diabetes | 2018 |
| 23261168 | The Southern European Atlantic Diet is associated with lower concentrations of markers of coronary risk. | Guallar-Castillon P, Oliveira A, Lopes C, Lopez-Garcia E, Rodriguez-Artalejo F. | Atherosclerosis | 2013 |
| 21736839 | Dietary patterns are associated with cardiometabolic risk factors in a representative study population of German adults. | Heidemann C, Scheidt-Nave C, Richter A, Mensink GB. | Br J Nutr | 2011 |
| 18574045 | Dietary patterns and risk of mortality from cardiovascular disease, cancer, and all causes in a prospective cohort of women. | Heidemann C, Schulze MB, Franco OH, van Dam RM, Mantzoros CS, Hu FB. | Circulation | 2008 |
| 30272853 | Dietary pattern and its association with blood pressure and blood lipid profiles among Japanese adults in the 2012 Japan National Health and Nutrition Survey. | Htun NC, Suga H, Imai S, Shimizu W, Ishikawa-Takata K, Takimoto H. | Asia Pac J Clin Nutr. | 2018 |
| 11010931 | Prospective study of major dietary patterns and risk of coronary heart disease in men. | Hu FB, Rimm EB, Stampfer MJ, Ascherio A, Spiegelman D, Willett WC. | Am J ClinNutr | 2000 |
| 19380370 | Dietary patterns and the risk of mortality: impact of cardiorespiratory fitness. | Heroux M, Janssen I, Lam M, Lee DC, Hebert JR, Sui X, Blair SN. | Int J Epidemiol | 2010 |
| 25898811 | Association between occupational psychosocial factors and waist circumference is modified by diet among men. | Jaaskelainen A, Kaila-Kangas L, Leino-Arjas P, Lindbohm ML, Nevanpera N, Remes J, Jarvelin MR, Laitinen J. | Eur J ClinNutr | 2015 |
| 31149710 | Generalizability of a Diabetes-Associated Country-Specific Exploratory Dietary Pattern Is Feasible Across European Populations. | Jannasch F, Kroger J, Agnoli C, Barricarte A, Boeing H, Cayssials V, Colorado-Yohar S, Dahm CC, Dow C, Fagherazzi G, Franks PW, Freisling H, Gunter MJ, Kerrison ND, Key TJ, Khaw KT, Kuhn T, Kyro C, Mancini FR, Mokoroa O, Nilsson P, Overvad K, Palli D, Panico S, Garcia JRQ, et al. | J Nutr. | 2019 |
| 25330000 | Dietary patterns differently associate with inflammation and gut microbiota in overweight and obese subjects. | Kong LC, Holmes BA, Cotillard A, Habi-Rachedi F, Brazeilles R, Gougis S, Gausserès N, Cani PD, Fellahi S, Bastard JP, Kennedy SP, Doré J, Ehrlich SD, Zucker JD, Rizkalla SW, Clément K. | PLoS One | 2014 |
| 27001278 | Dietary patterns in men and women are simultaneously determinants of altered glucose metabolism and bone metabolism. | Langsetmo L, Barr SI, Dasgupta K, Berger C, Kovacs CS, Josse RG, Adachi JD, Hanley DA, Prior JC, Brown JP, Morin SN, Davison KS, Goltzman D, Kreiger N. | Nutr Res | 2016 |
| 20109205 | Dietary patterns in Canadian men and women ages 25 and older: relationship to demographics, body mass index, and bone mineral density. | Langsetmo L, Poliquin S, Hanley DA, Prior JC, Barr S, Anastassiades T, Towheed T, Goltzman D, Kreiger N; CaMos Research Group.. | BMC MusculoskeletDisord | 2010 |
| 21865562 | Dietary patterns are associated with stroke in Chinese adults. | Li Y, He Y, Lai J, Wang D, Zhang J, Fu P, Yang X, Qi L. | J Nutr | 2011 |
| 19033409 | Food intake patterns associated with incident type 2 diabetes: the Insulin Resistance Atherosclerosis Study. | Liese AD, Weis KE, Schulz M, Tooze JA. | Diabetes Care | 2009 |
| 15447916 | Major dietary patterns are related to plasma concentrations of markers of inflammation and endothelial dysfunction. | Lopez-Garcia E, Schulze MB, Fung TT, Meigs JB, Rifai N, Manson JE, Hu FB. | Am J ClinNutr | 2004 |
| 24871477 | A provegetarian food pattern and reduction in total mortality in the Prevencia con Dieta Mediterranea (PREDIMED) study. | Martínez-González MA, Sánchez-Tainta A, Corella D, Salas-Salvadó J, Ros E, Arós F, Gómez-Gracia E, Fiol M, Lamuela-Raventós RM, Schröder H, Lapetra J, Serra-Majem L, Pinto X, Ruiz-Gutierrez V, Estruch R; PREDIMED Group. | Am J ClinNutr | 2014 |
| 21963016 | A meat, processed meat, and French fries dietary pattern is associated with high allostatic load in Puerto Rican older adults. | Mattei J, Noel SE, Tucker KL. | J Am Diet Assoc | 2011 |
| 19327192 | Food patterns associated with blood lipids are predictive of coronary heart disease: the Whitehall II study. | McNaughton SA, Mishra GD, Brunner EJ. | Br J Nutr | 2009 |
| 17182808 | Dietary patterns throughout adult life are associated with body mass index, waist circumference, blood pressure, and red cell folate. | McNaughton SA, Mishra GD, Stephen AM, Wadsworth ME. | J Nutr | 2007 |
| 22166270 | Factor analysis in the identification of dietary patterns and their predictive role in morbid and fatal events. | Menotti A, Alberti-Fidanza A, Fidanza F, Lanti M, Fruttini D. | Public Health Nutr | 2012 |
| 17367571 | Major dietary patterns and cardiovascular risk factors from childhood to adulthood. The Cardiovascular Risk in Young Finns Study. | Mikkilä V, Räsänen L, Raitakari OT, Marniemi J, Pietinen P, Rönnemaa T, Viikari J. | Br J Nutr | 2007 |
| 15671254 | Dietary patterns and the incidence of type 2 diabetes. | Montonen J, Knekt P, Härkänen T, Järvinen R, Heliövaara M, Aromaa A, Reunanen A. | Am J Epidemiol | 2005 |
| 23017319 | Effects of healthy dietary pattern and other lifestyle factors on incidence of diabetes in a rural Japanese population. | Morimoto A, Ohno Y, Tatsumi Y, Mizuno S, Watanabe S. | Asia Pac J ClinNutr | 2012 |
| 26244510 | A Snack Dietary Pattern Increases the Risk of Hypercholesterolemia in Northern Chinese Adults: A Prospective Cohort Study. | Na L, Han T, Zhang W, Wu X, Na G, Du S, Li Y, Sun C. | PLoS One | 2015 |
| 28445513 | Dietary patterns and all-cause, cancer, and cardiovascular disease mortality in Japanese men and women: The Japan public health center-based prospective study. | Nanri A, Mizoue T, Shimazu T, Ishihara J, Takachi R, Noda M, Iso H, Sasazuki S, Sawada N, Tsugane S; Japan Public Health Center-Based Prospective Study Group. | PLoS One | 2017 |
| 29807407 | Associations between dietary risk factors and ischemic stroke: a comparison of regression methods using data from the Multi-Ethnic Study of Atherosclerosis. | Nazari SSH, Mokhayeri Y, Mansournia MA, Khodakarim S, Soori H. | Epidemiol Health | 2018 |
| 18614740 | A priori-defined dietary patterns and markers of cardiovascular disease risk in the Multi-Ethnic Study of Atherosclerosis (MESA). | Nettleton JA, Schulze MB, Jiang R, Jenny NS, Burke GL, Jacobs DR Jr. | Am J ClinNutr | 2008 |
| 12791618 | Dietary patterns and changes in body mass index and waist circumference in adults. | Newby PK, Muller D, Hallfrisch J, Qiao N, Andres R, Tucker KL. | Am J ClinNutr | 2003 |
| 19458029 | A traditional rice and beans pattern is associated with metabolic syndrome in Puerto Rican older adults. | Noel SE, Newby PK, Ordovas JM, Tucker KL. | J Nutr | 2009 |
| 29625864 | Dietary Intakes of Branched-Chained Amino Acid and Risk for Type 2 Diabetes in Adults: The Harbin Cohort Study on Diet, Nutrition and Chronic Non-Communicable Diseases Study. | Okekunle AP, Wu X, Duan W, Feng R, Li Y, Sun C. | Can J Diabetes. | 2018 |
| 30377838 | Higher intakes of energy-adjusted dietary amino acids are inversely associated with obesity risk. | Okekunle AP, Wu X, Feng R, Li Y, Sun C. | Amino Acids. | 2019 |
| 17895884 | Three major dietary patterns are all independently related to the risk of obesity among 3760 Japanese women aged 18-20 years. | Okubo H, Sasaki S, Murakami K, Kim MK, Takahashi Y, Hosoi Y, Itabashi M; Freshmen in Dietetic Courses Study II group. | Int J Obes (Lond) | 2008 |
| 20484454 | Adherence to the Southern European Atlantic Diet and occurrence of nonfatal acute myocardial infarction | Oliveira,A. and Lopes,C. and Rodriguez-Artalejo,F. | American Journal of Clinical Nutrition | 2010 |
| 11242490 | Dietary patterns and mortality in Danish men and women: a prospective observational study. | Osler M, Heitmann BL, Gerdes LU, Jorgensen LM, Schroll M. | Br J Nutr | 2001 |
| 17524719 | The association between food patterns and the metabolic syndrome using principal components analysis: The ATTICA Study. | Panagiotakos DB, Pitsavos C, Skoumas Y, Stefanadis C. | J Am Diet Assoc | 2007 |
| 17350085 | Adherence to the Mediterranean food pattern predicts the prevalence of hypertension, hypercholesterolemia, diabetes and obesity, among healthy adults; the accuracy of the MedDietScore. | Panagiotakos DB, Pitsavos C, Arvaniti F, Stefanadis C. | Prev Med | 2007 |
| 17126772 | Dietary patterns: a Mediterranean diet score and its relation to clinical and biological markers of cardiovascular disease risk. | Panagiotakos DB, Pitsavos C, Stefanadis C. | NutrMetabCardiovasc Dis | 2006 |
| 29563039 | Dietary patterns, Mediterranean diet and obesity in postmenopausal women. | Papavagelis C, Avgeraki E, Augoulea A, Stamatelopoulos K, Lambrinoudaki I, Yannakoulia M. | Maturitas | 2018 |
| 19736556 | Associations between dietary patterns and obesity phenotypes. | Paradis AM, Godin G, Perusse L, Vohl MC. | Int J Obes (Lond) | 2009 |
| 27245103 | A high-fat, high-glycaemic index, low-fibre dietary pattern is prospectively associated with type 2 diabetes in a British birth cohort. | Pastorino S, Richards M, Pierce M, Ambrosini GL. | Br J Nutr | 2016 |
| 27299701 | Plant-Based Dietary Patterns and Incidence of Type 2 Diabetes in US Men and Women: Results from Three Prospective Cohort Studies. | Satija A, Bhupathiraju SN, Rimm EB, Spiegelman D, Chiuve SE, Borgi L, Willett WC, Manson JE, Sun Q, Hu FB. | PLoS Med | 2016 |
| 16988088 | Dietary patterns and changes in body weight in women. | Schulze MB, Fung TT, Manson JE, Willett WC, Hu FB. | Obesity (Silver Spring) | 2006 |
| 12915502 | Risk of hypertension among women in the EPIC-Potsdam Study: comparison of relative risk estimates for exploratory and hypothesis-oriented dietary patterns. | Schulze MB, Hoffmann K, Kroke A, Boeing H. | Am J Epidemiol | 2003 |
| 28032067 | Association of Major Dietary Patterns with Cardio-metabolic Risk Factors in Type 2 Diabetic Patients. | Shadman Z, Akhoundan M, Poorsoltan N, Larijani B, Qorbani M, Hedayati M, KhoshniatNikoo M. | Iran J Public Health | 2016 |
| 24966409 | Instant noodle intake and dietary patterns are associated with distinct cardiometabolic risk factors in Korea. | Shin HJ, Cho E, Lee HJ, Fung TT, Rimm E, Rosner B, Manson JE, Wheelan K, Hu FB. | J Nutr | 2014 |
| 29433580 | Dietary patterns and physical activity in the metabolically (un)healthy obese: the Dutch Lifelines cohort study. | Slagter SN, Corpeleijn E, van der Klauw MM, Sijtsma A, Swart-Busscher LG, Perenboom CWM, de Vries JHM, Feskens EJM, Wolffenbuttel BHR, Kromhout D, van Vliet-Ostaptchouk JV. | Nutr J | 2018 |
| 22647416 | Dietary patterns derived from principal component- and k-means cluster analysis: long-term association with coronary heart disease and stroke. | Stricker MD, Onland-Moret NC, Boer JM, van der Schouw YT, Verschuren WM, May AM, Peeters PH, Beulens JW. | NutrMetabCardiovasc Dis | 2013 |
| 29258212 | Dietary Patterns in Relation to Metabolic Syndrome among Adults in Poland: A Cross-Sectional Study. | Suliga E, Kozieł D, Cieśla E, Rębak D, Głuszek S. | Nutrients | 2017 |
| 26025375 | Association between dietary patterns and metabolic syndrome in individuals with normal weight: a cross-sectional study. | Suliga E, Kozieł D, Cieśla E, Głuszek S. | Nutr J | 2015 |
| 12716666 | Patterns of food consumption and risk factors for cardiovascular disease in the general Dutch population. | van Dam RM, Grievink L, Ocke MC, Feskens EJ. | Am J ClinNutr | 2003 |
| 11827496 | Dietary patterns and risk for type 2 diabetes mellitus in U.S. men. | van Dam RM, Rimm EB, Willett WC, Stampfer MJ, Hu FB. | Ann Intern Med | 2002 |
| 16046726 | A homocysteine metabolism-related dietary pattern and the risk of coronary heart disease in two independent German study populations. | Weikert C, Hoffmann K, Dierkes J, Zyriax BC, Klipstein-Grobusch K, Schulze MB, Jung R, Windler E, Boeing H. | J Nutr | 2005 |
| 10884714 | A cross-sectional study of dietary patterns with glucose intolerance and other features of the metabolic syndrome. | Williams DE, Prevost AT, Whichelow MJ, Cox BD, Day NE, Wareham NJ. | Br J Nutr | 2000 |
| 24095621 | Dietary patterns and total mortality in a Mediterranean cohort: the SUN project. | Zazpe I, Sánchez-Tainta A, Toledo E, Sánchez-Villegas A, Martínez-González MÁ. | J AcadNutr Diet | 2014 |

**Additional Table S4** List of 75 studies identified from abstract screening but excluded after full-text screening

| **PMID** | **Title** | **Author** | **Journal** | **Year** | **Reason for Exclusion** |
| --- | --- | --- | --- | --- | --- |
| 27353785 | Higher potato intake associated with hypertension risk. | [No authors listed] | Nurs Older People | 2016 | Not Design of interest |
| 26082846 | Dietary Patterns of Young Females and Their Association With Waist Circumference as a Health Index in Northwest of Iran, 2007. | Alizadeh M, Didarloo A, Esmaillzadeh A. | Iran Red Crescent Med J | 2015 | Included participants <18 years old |
| 30729640 | The relationship between pre-pregnancy dietary patterns adherence and risk of gestational diabetes mellitus in Iran: A case-control study. | Asadi M, Shahzeidi M, Nadjarzadeh A, Hashemi Yusefabad H, Mansoori A | Nutr Diet | 2019 | No outcome of interest |
| 28598991 | Change in potato consumption among Norwegian women 1998-2005-The Norwegian Women and Cancer study (NOWAC). | Attah AO, Braaten T, Skeie G. | PLoS One | 2017 | No outcome of interest |
| 25313577 | Comparison of two exploratory dietary patterns in association with the metabolic syndrome in a Northern German population. | Barbaresko J, Siegert S, Koch M, Aits I, Lieb W, Nikolaus S, Laudes M, Jacobs G, Nothlings U. | Br J Nutr | 2014 | No subgroup of interest |
| 31581416 | Eating Occasions, Obesity and Related Behaviors in Working Adults: Does it Matter When You Snack? | Barrington WE, Beresford SAA | Nutrients | 2019 | No association of interest reported |
| 28392166 | Legume consumption is inversely associated with type 2 diabetes incidence in adults: A prospective assessment from the PREDIMED study. | Becerra-Tomás N, Díaz-López A, Rosique-Esteban N, Ros E, Buil-Cosiales P, Corella D, Estruch R, Fitó M, Serra-Majem L, Arós F, Lamuela-Raventós RM, Fiol M, Santos-Lozano JM, Díez-Espino J, Portoles O, Salas-Salvadó J; PREDIMED StudyInvestigators. | ClinNutr | 2018 | Not design of interest |
| 25809805 | Nutrient patterns and brain biomarkers of Alzheimer's disease in cognitively normal individuals. | Berti V, Murray J, Davies M, Spector N, Tsui WH, Li Y, Williams S, Pirraglia E, Vallabhajosula S, McHugh P, Pupi A, de Leon MJ, Mosconi L. | J Nutr Health Aging | 2015 | No outcome of interest |
| 26394033 | Changes in Intake of Fruits and Vegetables and Weight Change in United States Men and Women Followed for Up to 24 Years: Analysis from Three Prospective Cohort Studies. | Bertoia ML, Mukamal KJ, Cahill LE,Hou T, Ludwig DS, Mozaffarian D, Willett WC,HuFB,RimmEB | PLoS Medicine | 2015 | No subgroup for potato |
| 3340176 | Diabetes and the potato. | Bittles AH. | Nature | 1988 | No association of interest reported |
| 24625460 | Associations between exposure to takeaway food outlets, takeaway food consumption, and body weight in Cambridgeshire, UK: population based, cross sectional study. | Burgoine T, Forouhi NG, Griffin SJ, Wareham NJ, Monsivais P. | BMJ | 2014 | No association of interest reported |
| 20573797 | Carbohydrate nutrition and inflammatory disease mortality in older adults. | Buyken AE, Flood V, Empson M, Rochtchina E, Barclay AW, Brand-Miller J, Mitchell P. | Am J ClinNutr | 2010 | No outcome of interest |
| 11237934 | Glycemic index in the diet of European outpatients with type 1 diabetes: relations to glycated hemoglobin and serum lipids. | Buyken AE, Toeller M, Heitkamp G, Karamanos B, Rottiers R, Muggeo M, Fuller JH; EURODIAB IDDM Complications Study Group.. | Am J ClinNutr | 2001 | No subgroup for potato |
| 15482612 | The UK Women's Cohort Study: comparison of vegetarians, fish-eaters and meat-eaters. | Cade JE, Burley VJ, Greenwood DC; UK Women's Cohort Study Steering Group. | Public Health Nutr | 2004 | No outcome of interest |
| 31393962 | The association of multimorbidity within cardio-metabolic disease domains with dietary patterns: A cross-sectional study in 129 369 men and women from the Lifelines cohort | Dekker LH, de Borst MH, Meems LMG, de Boer RA, Bakker SJL, Navis GJ. | PLoS One |  | No outcome of interest |
| 18326604 | Glycemic index and glycemic load in relation to food and nutrient intake and metabolic risk factors in a Dutch population. | Du H, van der A DL, van Bakel MM, van der Kallen CJ, Blaak EE, van Greevenbroek MM, Jansen EH, Nijpels G, Stehouwer CD, Dekker JM, Feskens EJ. | Am J ClinNutr | 2008 | No association of interest reported |
| 22578133 | Adherence to a Mediterranean diet in Morocco and its correlates: cross-sectional analysis of a sample of the adult Moroccan population. | El Rhazi K, Nejjari C, Romaguera D, Feart C, Obtel M, Zidouh A, Bekkali R, Gateau PB. | BMC Public Health | 2012 | No outcome of interest |
| 25969395 | Association between red and processed meat consumption and chronic diseases: the confounding role of other dietary factors. | Fogelholm M, Kanerva N, Männistö S. | Eur J ClinNutr | 2015 | No outcome of interest |
| 11093299 | Fast food restaurant use among women in the Pound of Prevention study: dietary, behavioral and demographic correlates. | French SA, Harnack L, Jeffery RW. | Int J ObesRelatMetabDisord | 2000 | No outcome of interest |
| 30947337 | Perspective: Refined Grains and Health: Genuine Risk, or Guilt by Association? | Gaesser GA | Adv Nutr | 2019 | No intervention of interest |
| none | Dietary patterns and weight loss in new-onset type 2 diabetes mellitus: a sub-analysis of the St Carlos study: a 3-year, randomized, clinic-based, interventional study | Garcia de la Torre N, Valle L, Duran A, Rubio MA, Fuentes M, Galindo M,Abad R, Sanz F, RunkleI,BarcaI,Calle-Pascual AL. | British Journal of Medicine and Medical Research | 2014 | No association of interest reported |
| 18503250 | Differences in overall mortality in the elderly may be explained by diet. | González S, Huerta JM, Fernández S, Patterson AM, Lasheras C. | Gerontology | 2008 | No outcome of interest |
| 30019088 | Improved adherence to Mediterranean Diet in adults with type 1 diabetes mellitus. | Granado-Casas M, Alcubierre N, Martin M, Real J, Ramirez-Morros AM, Cuadrado M, Alonso N, Falguera M, Hernandez M, Aguilera E, Lecube A, Castelblanco E, Puig-Domingo M, Mauricio D | Eur J Nutr | 2019 | No outcome of interest |
| none | Distribution and exposure prevalence of carbohydrate-based food intake among obese Korean adults based on the Health Examinees (HEXA) Study. [Korean] | Han YR, KwonSO, Lee SA. | Korean Journal of Community Nutrition | 2017 | No intervention of interest |
| 27275190 | Eating Habits and Lifestyles among a Sample of Obese Working Egyptian Women. | Hassan NE, Wahba SA, El-Masry SA, Elhamid ER, Boseila SA, Ahmed NH, Ibrahim TS. | Open Access Maced J Med Sci | 2015 | No intervention of interest |
| 16472386 | Dietary and other lifestyle correlates of serum folate concentrations in a healthy adult population in Crete, Greece: a cross-sectional study. | Hatzis CM, Bertsias GK, Linardakis M, Scott JM, Kafatos AG. | Nutr J | 2006 | No outcome of interest |
| 3622196 | Glycemic effects of spaghetti and potato consumed as part of mixed meal on IDDM patients. | Hermansen K, Rasmussen O, Arnfred J, Winther E, Schmitz O. | Diabetes Care | 1987 | No design of interest |
| No PMID | A comparison of elderly (20-65 yrs) life style with body mass index (BMI) < and >25 | Heydarnejad MS, Dehkordi AH | Journal of Hainan Medical College | 2010 | No association of interest |
| 31386561 | Type 2 diabetes prevalence among Canadian adults - dietary habits and sociodemographic risk factors | Hosseini Z, Whiting SJ, Vatanparast H. | Appl Physiol Nutr Metab | 2019 | No subgroup for population of interest |
| 26703721 | Assessing the Nutritional Quality of Diets of Canadian Adults Using the 2014 Health Canada Surveillance Tool Tier System. | Jessri M, Nishi SK, L'Abbe MR. | Nutrients | 2015 | No outcome of interest |
| 1877515 | Coronary-heart-disease risk-factor status of the Cretan urban population in the 1980s. | Kafatos A, Kouroumalis I, Vlachonikolis I, Theodorou C, Labadarios D. | Am J ClinNutr | 1991 | No association of interest reported |
| 6720674 | Association between reported diet and all-cause mortality. Twenty-one-year follow-up on 27,530 adult Seventh-Day Adventists. | Kahn HA, Phillips RL, Snowdon DA, Choi W. | Am J Epidemiol | 1984 | No outcome of interest |
| 22644320 | Dietary patterns in relation to bone mineral density among menopausal Iranian women. | Karamati M, Jessri M, Shariati-Bafghi SE, Rashidkhani B. | Calcif Tissue Int | 2012 | No outcome of interest |
| 28133993 | A "healthy diet-optimal sleep" lifestyle pattern is inversely associated with liver stiffness and insulin resistance in patients with nonalcoholic fatty liver disease. | Katsagoni CN, Papatheodoridis GV, Papageorgiou MV, Ioannidou P, Deutsch M, Alexopoulou A, Papadopoulos N, Fragopoulou E, Kontogianni MD. | ApplPhysiolNutrMetab | 2017 | No potato data |
| none | The association between dietary patterns and bone mineral density in a sample of postmenopausal women living in Tehran. [Persian] | Keramati M, Bafghi ES, Rajaii AR, Rashidkhani B. | Iranian Journal of Nutrition Sciences & Food Technology | 2012 | Foreign Language- unable to translate |
| none | Relationship between food and nutrient intake and the risk of hypertriglyceridemia in Vietnamese women residing in Bavi: the Korean Genome and Epidemiology Study (KoGES). [Korean] | KimJK, Kim JM, Kim HS, Chung HW, Chang NS. | Korean Journal of Nutrition | 2013 | No potato data |
| 17187681 | Reported food intake and distribution of body fat: a repeated cross-sectional study. | Krachler B, Eliasson M, Stenlund H, Johansson I, Hallmans G, Lindahl B. | Nutr J | 2006 | No potato data |
| 22027052 | Young adults and eating away from home: associations with dietary intake patterns and weight status differ by choice of restaurant. | Larson N, Neumark-Sztainer D, Laska MN, Story M. | J Am Diet Assoc | 2011 | No association of interest reported |
| 23599238 | Fruit and vegetable consumption and mortality: European prospective investigation into cancer and nutrition. | Leenders M, Sluijs I, Ros MM, Boshuizen HC, Siersema PD, Ferrari P, Weikert C, Tjønneland A, Olsen A, Boutron-Ruault MC, Clavel-Chapelon F, Nailler L, Teucher B, Li K, Boeing H, Bergmann MM, Trichopoulou A, Lagiou P, Trichopoulos D, Palli D, Pala V, Panico S, Tumino R, Sacerdote C, Peeters PH, van Gils CH, Lund E, Engeset D, Redondo ML, Agudo A, Sánchez MJ, Navarro C, Ardanaz E, Sonestedt E, Ericson U, Nilsson LM, Khaw KT, Wareham NJ, Key TJ, Crowe FL, Romieu I, Gunter MJ, Gallo V, Overvad K, Riboli E, Bueno-de-Mesquita HB. | Am J Epidemiol | 2013 | No intervention of interest |
| none | Relationship of dietary pattern with obesity, hypertension and diabetes in Chinese men in Jilin city. [Chinese] | Li SJ, Liu WS, BaiXS. | Chinese Journal of Prevention and Control of Chronic Diseases | 2011 | No subgroup for potato |
| 29949199 | Lean non-alcoholic fatty liver disease patients had comparable total caloric, carbohydrate, protein, fat, iron, sleep duration and overtime work as obese non-alcoholic fatty liver disease patients. | Li C, Guo P, Okekunle AP, Ji X, Huang M, Qi J, Jiang Y, Feng R, Li R | J Gastroenterol Hepatol | 2019 | Not population of interest |
| 17125525 | Specific food intake, fat and fiber intake, and behavioral correlates of BMI among overweight and obese members of a managed care organization. | Linde JA, Utter J, Jeffery RW, Sherwood NE, Pronk NP, Boyle RG. | Int J BehavNutrPhys Act | 2006 | No design of interest |
| 22575037 | Candidate measures of whole plant food intake are related to biomarkers of nutrition and health in the US population (National Health and Nutrition Examination Survey 1999-2002). | Lipsky LM, Cheon K, Nansel TR, Albert PS. | Nutr Res | 2012 | Included participants <18 years old |
| 29756972 | Dietary patterns, physical activity, body mass index, weight-related behaviours and their interrelationship among Romanian university students-trends from 2003 to 2016 | Lotrean LM, Stan O, Codruta L, Laza V. | Nutr Hosp | 2018 | No outcome of interest |
| 29753713 | Weight gain in first-semester university students: Positive sleep and diet practices associated with protective effects. | Ludy MJ, Tan SY, Leone RJ, Morgan AL, Tucker RM. | PhysiolBehav | 2018 | No intervention of interest |
| 24661608 | Trends in dietary carbohydrate consumption from 1991 to 2008 in the Framingham Heart Study Offspring Cohort. | Makarem N, Scott M, Quatromoni P, Jacques P, Parekh N. | Br J Nutr | 2014 | No outcome of interest |
| none | Multifactorial analysis of dietary patterns in healthy and coronary artery disease patients: brief report. [Persian] | Maleki A, Ashjaearvan M, KarimiA. | Tehran University Medical Journal | 2015 | Foreign Language- unable to translate |
| 31185656 | Maternal Dietary Patterns Are Associated with Pre-Pregnancy Body Mass Index and Gestational Weight Gain: Results from the "Mamma & Bambino" Cohort | Maugeri A, Barchitta M, Favara G, La Rosa MC, La Mastra C, Magnano San Lio R, Agodi A. | Nutrients | 2019 | Not population of interest |
| 30994167 | The impact of social determinants and lifestyles on dietary patterns during pregnancy: evidence from the 'Mamma & Bambino' study. | Maugeri A, Barchitta M, Agrifoglio O, Favara G, La Mastra C, La Rosa MC, Magnano San Lio R, Panella M, Cianci A, Agodi A | Ann Ig | 2019 | Not population of interest |
| 28247963 | Nutrition and physical activity transitions in the Ecuadorian Andes: Differences among urban and rural-dwelling women. | Melby CL, Orozco F, Ochoa D, Muquinche M, Padro M, Munoz FN. | Am J Hum Biol | 2017 | No outcome of interest |
| 28112076 | Energy density of the diets of Japanese adults in relation to food and nutrient intake and general and abdominal obesity: a cross-sectional analysis from the 2012 National Health and Nutrition Survey, Japan. | Murakami K, Livingstone MB, Okubo H, Sasaki S. | Br J Nutr | 2017 | No outcome of interest |
| 24012281 | Serum adiponectin concentration in relation to macronutrient and food intake in young Japanese women. | Murakami K, Sasaki S, Uenishi K; Japan Dietetic Students’ Study for Nutrition and Biomarkers Group. | Nutrition | 2013 | No outcome of interest |
| 30080215 | Dietary Patterns and Retinal Vessel Caliber in the Irish Nun Eye Study. | Neville CE, Montgomery S, Silvestri G, McGowan A, Moore E, Silvestri V, Cardwell C, McEvoy CT, Maxwell AP, Woodside JV, McKay GJ | J Nutr Health Aging | 2018 | No association of interest reported |
| 917137 | [Follow-up studies after 7 years of the risk factors for coronary disease in young managers]. | Nieveen J, May JF, Burema J, Distelbrink CA, Kwarts E, Mulder HG. | Ned TijdschrGeneeskd | 1977 | No association of interest reported |
| 29806862 | Potato Consumption Is not Associated with Higher Risk of Mortality: A Longitudinal Study among Southern Italian Older Adults. | Osella AR, Veronese N, Notarnicola M, Cisternino AM, Misciagna G, Guerra V, Nitti A, Campanella A, Caruso MG. | J Nutr Health Aging | 2018 | No outcome of interest |
| 22548847 | Ageing, arterial blood pressure, body mass index, and diet. | Pavlović M, Milković-Kraus S, Jovanović V, Hercigonja-Szekeres M. | ArhHig Rada Toksikol | 2012 | No outcome of interest |
| 19936480 | [Changing in dietary intake by women in the Municipality of Rio de Janeiro, Brazil, from 1995 to 2005]. | Pereira RA, Andrade RG, Sichieri R. | Cad SaudePublica | 2009 | No outcome of interest |
| 12571653 | Low prevalence of the metabolic syndrome in wine drinkers--is it the alcohol beverage or the lifestyle? | Rosell M, De Faire U, Hellenius ML. | Eur J Clin Nutr | 2003 | No outcome of interest |
| 2798367 | Lifestyle and mortality among Norwegian men. | Rotevatn S, Akslen LA, Bjelke E. | Prev Med | 1989 | No outcome of interest |
| 28728684 | Healthful and Unhealthful Plant-Based Diets and the Risk of Coronary Heart Disease in U.S. Adults. | Satija A, Bhupathiraju SN, Spiegelman D, Chiuve SE, Manson JE, Willett W, Rexrode KM, Rimm EB, Hu FB. | J Am Coll Cardiol | 2017 | No potato data |
| 2613381 | Similarities and differences between the epidemiology and possible dietary causes of coronary arterial disease and strokes. | Seely S. | Int J Cardiol | 1989 | No potato data |
| 31382881 | Sex-related differences in the association between frailty and dietary consumption in Japanese older people: a cross-sectional study. | Shibasaki K and Kin SK and Yamada S and Akishita M and Ogawa S | BMC Geriatr | 2019 | No outcome of interest |
| 29770760 | Adherence to the New Nordic Diet during pregnancy and subsequent maternal weight development: a study conducted in the Norwegian Mother and Child Cohort Study (MoBa). | Skreden M and Hillesund ER and Wills AK and Brantsaeter AL and Bere E and Overby NC | Br J Nutr | 2018 | Not population of interest |
| 3228821 | Age retirement in women. II. Dietary habits and body composition. | Steen B, Nilsson K, Robertsson E, Ostberg H. | ComprGerontol A | 1988 | No potato data |
| 24642783 | Nutrient-rich foods, cardiovascular diseases and all-cause mortality: the Rotterdam study. | Streppel MT, Sluik D, van Yperen JF, Geelen A, HofmanA, Franco OH, Witteman JC, Feskens EJ. | Eur J ClinNutr | 2014 | No association of interest reported |
| none | Association between the metabolic syndrome and food patterns in non-menopause women. [Persian] | Tabrizi SH, FarajiMH,Rad AH, Abadi A, HosseinpanahF. | Iranian Journal of Nutrition Sciences & Food Technology | 2010 | Foreign Language- unable to translate |
| 28885553 | Deviation of Chinese Adults' Diet from the Chinese Food Pagoda 2016 and Its Association with Adiposity. | Tian X, Huang Y, Wang H. | Nutrients | 2017 | No potato subgroup |
| 16502948 | Vegetable and fruit intake and mortality from chronic disease in New Zealand. | Tobias M, Turley M, Stefanogiannis N, Vander Hoorn S, Lawes C, Mhurchu CN, Rodgers A. | Aust N Z J Public Health | 2006 | No intervention of interest |
| 15851646 | Mediterranean diet and survival among patients with coronary heart disease in Greece. | Trichopoulou A, Bamia C, Trichopoulos D. | Arch Intern Med | 2005 | No outcome of interest |
| none | Prevalence of obesity, food consumption pattern and dietary intake of working women in B.H.U, Varanasi | Upasana and Archana,Chakravarty | Food Science Research Journal | 2015 | No association of interest |
| 29509665 | Generic Meal Patterns Identified by Latent Class Analysis: Insights from NANS (National Adult Nutrition Survey). | Uzhova I, Woolhead C, Timon CM, O'Sullivan A, Brennan L, Penalvo JL, Gibney ER | Nutrients | 2018 | No potato subgroup |
| 29911312 | Cross-sectional analysis of unhealthy foods, race/ethnicity, sex and cardiometabolic risk factors in U.S. adults | Vaccaro JA., Zarini GG., Huffman FG. | Nutr Diet. | 2018 | No association of interest reported |
| 28592612 | Fried potato consumption is associated with elevated mortality: an 8-y longitudinal cohort study. | Veronese N, Stubbs B, Noale M, Solmi M, Vaona A, Demurtas J, Nicetto D, Crepaldi G, Schofield P, Koyanagi A, Maggi S, Fontana L. | Am J ClinNutr | 2017 | No outcome of interest |
| 32344473 | [Influence of dietary patterns on type 2 diabetes mellitus in local residents aged 40 years and above in Songjiang district, Shanghai] | Zhu WL, Guan Y, Xu CZ, Liu ZX, Zhao GM, Jiang YG, Wang WB | Zhonghua Liu Xing Bing Xue Za Zhi | 2020 | Foreign Language |
| 25880233 | Dietary antioxidant capacity of the patients with cardiovascular disease in a cross-sectional study. | Zujko ME, Witkowska AM, WaÅ›kiewicz A, Piotrowski W, Terlikowska KM. | Nutr J | 2015 | No potato subgroup |

**Additional Table S5** Baseline characteristics of included studies

| Author, Year | Cohort Name | Study Design | Enroll-ment Year(s) (Total Follow-up) | Country (Funding) | N Analyzed | Reported age | % Males | Baseline Health Status* | Potato type | Analysis type  (Subgroups reported) | Intake amounts | Outcome reported |
| --- | --- | --- | --- | --- | --- | --- | --- | --- | --- | --- | --- | --- |
|  |  |  |  |  |  |  |  |  |  |  |  |  |
| Pietinen 1996 8941095 | Alpha-Tocopherol, Beta-Carotene Cancer Prevention Study | Cohort | 1985-1988 (6.1 years) | Finland  (Gov) | 21,930 | 57 | 100 | Mixed | Potatoes | Quintile  (NA) | 95.3, 135.3, 168.6, 209.6, 286.5 g/  day | Coronary mortality; Quantitative: Major Coronary Events |
| Masala 2008 18854749 | EPIC Florence | Cross-sectional | 1993-1998 (NA) | Italy  (Mixed) | 7,601 | 50.7 | 0 | Overweight, obese | Potatoes | Linear  (NA) | 50 g increment | DBP, SBP |
| Dilis 2012 22894912 | EPIC- Greek | Cohort | 1994-1999 (229,895 py) | Greece  (Mixed) | 23,929 | 20-86 | 40.7 | Mixed | Potatoes | Linear (Men, Women) | per 1 g/day standard deviation increment | CHD mortality, CHD incidence |
| von Ruesten 2013 23388667 | EPIC Potsdam | Cohort | 1994-1998 (8 years) | Germany  (Mixed) | 23,531 | 35-65 | 38.7 | Healthy | Potatoes, French fries | Linear (Potatoes, French fries) | Each 100 g/day increase | CVD, Diabetes |
| Tormo 2000 11338129 | The Spanish EPIC cohort | Cross-sectional | 1992-1996 (NA) | Spain  (Gov) | 41,391 | 49.9 | 37.7 | Hypertension | Potatoes | Case-control  (Men, Women) | 87.1 vs. 87.8 g (M), 64.0 vs. 61.9 g (W) | Blood pressure |
| Montonen 2005 15674312; Mizrahi 2009 19646291 | Finnish Mobile Clinic Health Examination Survey | Cohort | 1967-1972 (24 years) | Finland  (Gov) | 4,304 | 51.9 | 53.1 | Mixed | Potatoes | Quintile (NA) | <132, 132-196, 197-283, >283 g/day | Type 2 Diabetes; Strokes |
| Han 2016 27851788 | HDNNCDS | Cross-sectional | NR  (NA) | China  (Mixed) | 8,764 | 49.6 | 35 | Mixed | Potatoes | Linear (NA) | 50 g/d increase | Type 2 Diabetes |
| French 1994 8186811 | Healthy Worker Project | Cohort | NR  (2 years) | USA  (Gov) | 3,552 | 38.1 | 46.1 | NR | French fries | Linear (NA) | One serving/  week increase | Body weight |
| Kim 2000 No PMID | Korean adult population in Jeonju | Cross-sectional | 1994  (NA) | South Korea  (Gov) | 110 | 59.4 | 39.1 | NR | Potatoes | Linear (NA) | 1 g/day increase | FBG, SBP, DBP, TC, BMI |
| Sonestedt 2015 25898210 | Malmo Diet and Cancer Study | Cohort | 1991-1996 (14 years) | Sweden  (Mixed) | 26,445 | 57.9 | 37.5 | NR | Potatoes | Case-control, Linear, Quintile (1991-2009 cohort; 1992-1994 cohort) | 45, 86, 115, 147, 212 g;  1 g/day increase | CVD incidence, coronary event, DBP, SBP, TG, HDL, LDL, HOMA |
| Hashemian 2019 31063480 | NIH-AARP | Cohort | 1995-2011  (15.6 years) | USA  (Gov) | 410,701 | 62.2 | 56.9 | No heart disease | Mixed | Linear | 1 serving increase/day | CHD mortality |
| Khosravi-Boroujeni 2013 23432170 | NR | Case-control | 2008  (NA) | Iran  (Aca) | 390 | 64.7 | 53.1 | Overweight, obese | Boiled | Quintile (NA) | 5.3, 16.2, 26.0, 60.0 g/day | Stroke |
| Lasheras 2003 14576712 | NR | Cross-sectional | NR  (NA) | Spain  (NR) | 162 | 73.7 | 48.1 | Healthy | Boiled and fried | Linear, Quintile (NA) | <21.6 21.6-29.7, 29.8-60.5, >60.5 g/d;  per 10 gram change | MDA |
| Tavani 2003 12807839 | NR | Case-control | 1976  (NA) | Italy  (Gov) | 881 | 60 | 68.5 | CVD | Cooked or mashed | Tertile (NA) | 1.1, 2.0, NR servings/ week | MI |
| Hu 2017 29046405; Konieczna 2019 31882021 | PREDIMED | Cohort | 2003-2009 (5 years) | Spain  (Gov) | 6,940; 7009 | 67 | 42.7 | Mixed | Mixed | Linear; Quintile (potato chips, French fries, boiled potato) | per 1 serving increase; 24.9, 52.6, 87.4, 102.3, 142.5 g/day | SBP, DBP, Hypertension; weight, waist circumference |
| Hu 2017 29046405 | SUN | Cohort | 1999-NR  (6.7 years) | Spain  (Gov) | 13,837 | 36 | 36 | Healthy | Mixed | Quintile (French fries, boiled potato, potato chips) | 11.4, 30.1, 45.3, 68.1, 108.7 g/day | Hypertension incident |
| Farhadnejad 2018 29909965 | Tehran Lipid and Glucose Study | Cohort | 2006-2015  (6 years) | Iran  (Aca) | 1,981 | 38.9 | 46.2 | Mixed | Mixed | Quartile  (boiled potato, fried potato) | 7.3, 16.05, 29.22, 50.50 g/day | Type 2 Diabetes |
| De Meyer 2018 29695838 | The Asklepios Study | Cross- sectional | 2004-NR  (NA) | Belgium  (Mixed) | 2,590 | 46 | 48.5 | Healthy | Fried potato | Linear  (NA) | per 100 g/day increase | BMI, hs-CRP, oxLDL |
| Wurtz 2016 27774916 | The Diet, Cancer and Health study | Cohort | 1993-1997 (13.6 years) | Denmark  (Mixed) | 55,171 | 56 | 47.2 | Mixed | Not potato chips | Linear (Men, Women) | per 150 g/week increase | Myocardial Infarction |
| Ruttgers 2015 26439793 | The PopGen Control cohort | Cross-sectional | 2005-2006 (NA) | Germany  (Gov) | 585 | 60.8 | 58.8 | NR | Potatoes | Linear (NA) | 1 g/d increase | VAT, SAAT |
| Sharma 2015 27930484 | The Shape Up Houston Texas Medical Center Evaluation Study | Cross-sectional | 2012  (NA) | USA  (Mixed) | 924 | 43.6 | 14.5 | Overweight, obese | Fried potato, Not Fried | Linear (Fried potato, Not Fried) | 1 cup increase/month | BMI classification |
| Liu 2004 15562224 | Women's Health Study | Cohort | 1993  (8.8 years) | USA  (NR) | 38,018 | >44 | 0 | Overweight, obese | NR | Quintile (NA) | 0.13, 0.28, 0.43, 0.56, 0.93 servings/day | Type 2 Diabetes |
| Muraki 2016 26681722; Joshipura 1999 10517425; Borgi 2016 27189229 | HPFS | Cohort | 1986  (14 years) | USA  (Gov) | 40,669 | 52.9 | 100 | Mixed | Mixed | Linear, Quartile, Quintile (Fried; Baked) | < 1, 1, 2-4, 5-6, >=7 servings/week; One serving/  day increase | Stroke; Type 2 Diabetes; Hypertension |
| Ascherio 1996 8621198; Joshipura 1999 10517425; Muraki 2016 26681722; Borgi 2016 27189229; Halton 2006 16469985 | NHS | Cohort | 1976  (20 years) | USA  (Gov) | 84,555 | 56 | 0 | Mixed | Mixed | Linear, Quintile (Baked; Chips; Fried; Baked, Boiled, or Mashed) | < 1, 1, 2-4, 5-6, >6 servings/week; One serving/  day increase | SBP, DBP (bad chips, good baked), Hypertension, Stroke, Diabetes |
| Shimakawa 1993 8269793 |  | Case-control |  |  | 2,658 | 53.3 | 0 | Mixed | Cooked or mashed | Case-control (NA) | 0.35 vs. 0.32 cup/day | Diabetes |
| Muraki 2016 26681722; Borgi 2016 27189229 | NHS II | Cohort | 1989  (20 years) | USA  (Gov) | 88,475 | 36.1 | 0 | Mixed | Mixed | Quartile, Quintile (Baked, boiled, mashed) | <1/month, 1-3/  month,  1-3/  week, >=4/  week | Diabetes, Hypertension |
| *Joshipura 2001 11412050* | *NHS, HPFS Combined* | *Cohort Combined* | *1976, 1986 (744854 py)* | *USA*  *(Gov)* | *126,399* | *30-75* | *33* | *Mixed* | *Potatoes* | *Linear (NA)* | *1 serving/*  *day increase* | *CHD* |
| *Mozaffarian 2011 21696306* | *NHS, NHS II, HPFS Combined* | *Cohort Combined* | *1986, 1991, 1986* | *USA*  *(Gov)* | *120,877* | *46.1* | *18.7* | *Mixed* | *Potatoes, potato chips* | *Linear (French fries; Boiled, baked, or mashed)* | *1 serving/*  *day increase* | *Weight change* |

Aca: Academia; BMI: Body Mass Index; Gov: Government; g: grams; HDNNCDS: Harbin Cohort Study on Diet, Nutrition and Chronic Non communicable Disease; HPFS: Health Professionals Follow-up Study; hs-CRP: High-sensitivity C-reactive Protein; MI: Myocardial infarction; MS: Metabolic Syndrome; NA: Not Applicable; NHS: Nurses’ Health Study; NHS II: Nurses' Health Study II; NIH-AARP: National Institute of Health- American Association of Retired Persons; oxLDL: Oxidized low-density lipoprotein; PREDIMED: Prevención con Dieta Mediterránea; SUN (Seguimiento Universidad de Navarra); USA: United States of America

*Mixed population consists of a population with a least two of the following: obese, overweight, diabetes, hypertension, healthy, hyperlipidemia, metabolic syndrome
